# *FineMAV*: prioritizing candidate genetic variants driving local adaptations in human populations

**DOI:** 10.1186/s13059-017-1380-2

**Published:** 2018-01-17

**Authors:** Michał Szpak, Massimo Mezzavilla, Qasim Ayub, Yuan Chen, Yali Xue, Chris Tyler-Smith

**Affiliations:** 1 Wellcome Trust Sanger Institute, Wellcome Genome Campus, Hinxton, CB10 1SA UK; 2 Division of Experimental Genetics, Sidra Medical and Research Center, Doha, Qatar; 3 Present Address: Genomics Facility, School of Science, Monash University Malaysia, Bandar Sunway, Selangor, Darul Ehsan Malaysia

**Keywords:** Human evolution, Positive selection, Selective sweep, Local adaptation, *FineMAV*

## Abstract

We present a new method, Fine-Mapping of Adaptive Variation (*FineMAV*), which combines population differentiation, derived allele frequency, and molecular functionality to prioritize positively selected candidate variants for functional follow-up. We calibrate and test *FineMAV* using eight experimentally validated “gold standard” positively selected variants and simulations. *FineMAV* has good sensitivity and a low false discovery rate. Applying *FineMAV* to the 1000 Genomes Project Phase 3 SNP dataset, we report many novel selected variants, including ones in *TGM3* and *PRSS53* associated with hair phenotypes that we validate using available independent data. *FineMAV* is widely applicable to sequence data from both human and other species.

**Electronic supplementary material:**

The online version of this article (10.1186/s13059-017-1380-2) contains supplementary material, which is available to authorized users.

## Background

The out-of-Africa expansion ~ 60,000 years ago exposed humans to a diverse range of new environments and selective pressures including new pathogens, climatic conditions, and diets [1–3]. Genetic drift and local adaptations in spatially distant populations consequently led to geographically structured phenotypic diversification, illustrated by the inter-population variation observed for numerous morphological and physiological traits, such as skin pigmentation [2–4]. Not only are the genetic variants underlying differences between populations crucial for understanding recent human evolution and present-day human diversity, but they may also be clinically relevant, as the prevalence and susceptibilities of some common diseases vary across regions (e.g. hypertension or type 2 diabetes) [4–6]. Medical implications of adaptive variation arise because natural selection can only act in a direct way on functionally important variants driving phenotypic variation [7, 8]; selected alleles usually confer protective effects, like pathogen resistance associated with *CASP12* [9], *CCR5* [10], and *FUT2* [11] deficiency alleles, but paradoxically, may turn harmful in non-traditional environments or a homozygous state [5, 6, 12–14], e.g. sickle cell alleles [15], *CPT1A* [16, 17], and *APOL1* [18, 19].

Selective episodes leave signatures in the human genome and thus can be recognized from the pattern of nucleotide polymorphisms in a population sample [2, 20, 21]. Most methods that have been developed to detect signals of recent and ongoing positive selection are based on the classical hard sweep model [2, 22]. This model assumes that a new advantageous mutation rapidly spreads to fixation or high frequency, purging nearby linked variation due to genetic hitchhiking [2, 20, 23]. Its genetic characteristics include high-frequency derived long-range haplotypes with a concomitant reduced level of genetic variation, large derived allele frequency differences between populations, and changes to the allele frequency spectrum (e.g. increased fraction of derived common and rare alleles, depletion of intermediate-frequency variation), although these features can also arise by genetic drift or purifying selection and are confounded by population demography [2–4, 7, 20, 22]. However, it has been argued that hard sweeps were rather rare in recent human evolution [2, 22] and that selection may more often operate on pre-existing variation that has evolved neutrally in the population until it becomes advantageous under certain conditions (“selection on standing variation”) [2, 4, 22]. Selection from standing variation is difficult to detect using most standard approaches, because the selected variant often exists on multiple haplotype backgrounds (a so-called “soft sweep”) and has weaker effects on closely linked sites, so does not produce the classical selective sweep signatures of extended linkage disequilibrium (LD) and site frequency spectrum (SFS) changes [2, 4, 21, 22, 24, 25].

Previous surveys have reported vast lists of putatively selected genomic segments, genes, and variants, which contrast sharply with the handful of functionally validated examples of genetic adaptations with both a strong population selection signal and a compelling explanation for the reasons for selection linked to a relevant phenotype in humans [2, 5, 22, 26]. This is because population-genetic-based methods are often imprecise, implicating large genomic regions harboring many genes and a myriad of single nucleotide polymorphisms (SNPs) that could potentially drive the selection signal, but which are mostly neutral [27]. Even if a selection statistic operates at the individual variant level, such as population-differentiation-based statistics (e.g. *F*_ST_; difference in derived allele frequency [*ΔDAF*]) [28] or some composite likelihood approaches (e.g. composite of multiple signals [*CMS*]) [29], the highest scoring variant is not necessarily causal. High LD around the selected SNP often results in a stretch of highly differentiated variants with the same allele frequencies, further complicating the identification of the most likely causal variant. Similarly, for each potentially causal variant identified by *CMS*, there are on average 20 neutral proxies, all indistinguishable from the functional mutation [29]. As a result, the false discovery rate (FDR) of genome-wide selection scans is potentially high, which is reflected by the low concordance between such studies [2, 6, 7, 22, 26, 30–32]. The focus of this field now needs to move from candidate locus discovery to fine mapping of the signals of selection and biological understanding of their adaptive significance. However, population genetics alone is usually not sufficient to narrow down the signal of selection to a single causative SNP and the only way to distinguish true positives from artifacts or neutral passenger variation has been functional validation [2, 33]. Yet very few variants have been validated in this way, as current technology does not allow high-throughput functional validation, e.g. using genome editing in model systems [33]. Therefore, a useful step would be to subject candidate variants to rigorous evaluation and narrow down these extensive lists to a manageable subset of the strongest candidates for functional studies.

Despite these reservations, there are a few well-supported cases of local genetic adaptation that conform to the classical sweep model [22]. One example is the A allele at rs1426654 (within *SLC24A5*), which is nearly fixed in European populations, causing an amino acid (Thr to Ala) change and contributing to lighter skin pigmentation [34]. Such examples are not restricted to amino acid changes and have also been reported for cis-regulatory variants, such as the A allele at the rs4988235, an intronic regulatory variant in *MCM6* which has been shown to increase the expression of the downstream lactase (*LCT*) gene in vitro enabling digestion of the milk sugar, lactose, as an adult in West Asian and European populations that traditionally practice pastoralism [35, 36].

**Fig. 1:**
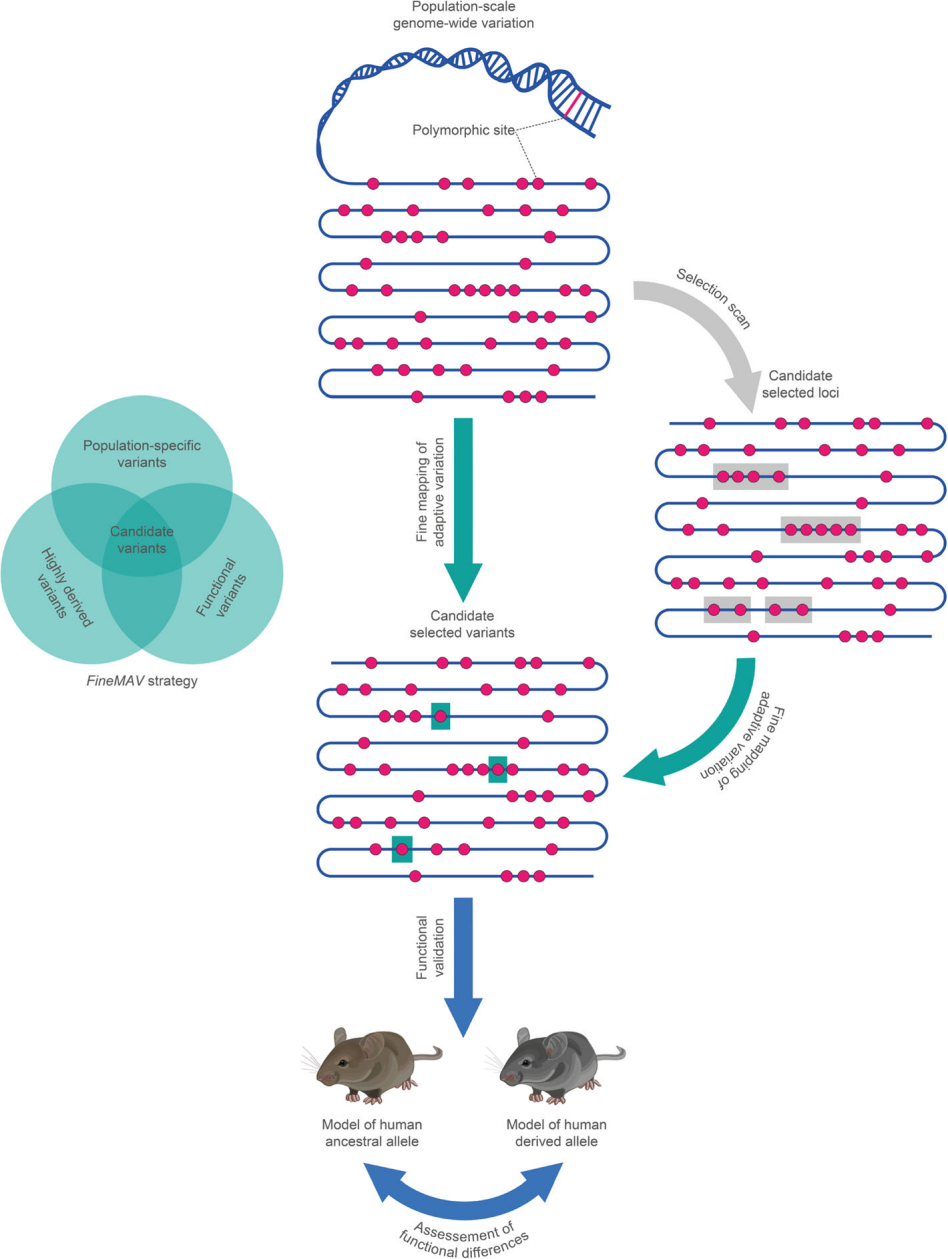
Workflow for prioritization of positively selected candidate variants for functional studies. The DNA molecule is represented as a *blue line*, with variants being *red dots*. Identification of the candidate positively selected variants from the genome-wide variation data, or the refinement of the known signal of selection to a functional SNP, is achieved by overlapping the statistical support from genetic analyses with functional annotation (implemented in *FineMAV*). A detailed follow-up functional study can then be performed (in vitro or in vivo experiments using model systems) to validate the implicated variant, quantify its phenotypic consequences, and clarify its relationship with reproductive fitness, e.g. by assessment of phenotypic differences between mouse models carrying the human-selected and non-selected alleles

Here, we develop a new in silico framework to shortlist candidate positively selected variants for further functional follow-up (Fig. 1). In order to prioritize candidate variants, we need a starting list of variants, a protocol for prioritization, and a way of assessing whether or not the prioritization is effective. We use an integrative method that overlays population signatures of selection with functional annotation to produce a refined list of candidate variants in the 1000 Genomes Project Phase 3 SNP dataset [37]. We assessed the results using both eight “gold standard” examples where the evidence for positive selection acting on a particular variant is convincing; and also simulations, to explore the likely false positive and negative rates and further discuss some of the novel variants in our lists.

## Results

We performed a new analysis of 1000 Genomes Project Phase 3 whole-genome sequence data [37] focusing on identifying individual putatively selected SNPs. Our analysis overlays multiple lines of evidence for causality to prioritize the vast numbers of potential candidates in order to identify a small number for experimental follow up. *FineMAV* combines a new measure of population differentiation (derived allele purity [*DAP*]; see “Methods” Eq. 2), a measure of allele prevalence (*DAF*), and a measure of functionality (the Combined Annotation-Dependent Depletion [*CADD*] PHRED-scaled C-score [38]). We simply scaled and combined them to obtain a single measure giving high values to derived alleles that are common, population-specific, and functional (see “Methods” Eq. 1). *FineMAV* is designed to refine the location of a positive selection signal to a single variant and can be applied to a region of prior interest or to the whole genome for de novo discovery of selected variants, focusing on recent local adaptations, that arose after the out-of-Africa population expansion.

### *FineMAV* power analyses using simulations

*FineMAV*’s power to detect selected variants depends on the strength of the selection coefficient. In simulations, it was unable to distinguish weak selection (s = 0.001) from neutrality since population differentiation under the scenarios tested was low (Additional file 1: Figure S1). In contrast, medium and strong selection coefficients (s = 0.007 and s = 0.01) produced *FineMAV* distributions that were different from the neutral variation (Additional file 1: Figure S1) and, assuming that *CADD* annotation is characterized by a low FDR, it was rare to find neutral variants in the extreme upper tail of the *FineMAV* distribution: FDR ~ 4%. The power to detect the selected variants that fall outside of the neutral *FineMAV* distribution was 46% and 77% for s = 0.007 and s = 0.01, respectively, although the real power, which depends on the accuracy of the functional annotation, might be lower (since functional annotation might be incomplete), these simulations demonstrated that *FineMAV* fits our aims, as we do not attempt to pick up all positive selection in the genome (accepting a high false negative rate), but rather try to minimize the FDR, which was < 5%.

### *FineMAV* evaluation using 1000 Genomes Project data

To calibrate *FineMAV* and evaluate its performance, we compiled a gold standard panel of the eight best examples of experimentally validated, positively selected variants underlying signals of positive selection that are linked to specific phenotypic consequences in the three well-characterized continental populations (Table 1). A key element was the value of the penalty for allele sharing between populations (parameter *x*). We first learned *x* from empirical data (subsets of the gold standards) and then tested it using simulations (100 simulated positive controls) to see if further increment of *x* increased *FineMAV*’s robustness in larger datasets (see “Methods”). The simulations showed that *x* deduced from empirical data was sufficient to pick up simulated selected variants and that its further increase did not affect *FineMAV*’s power. Calibration results were consistent across different combinations of gold standards used in the analysis (see “Methods”). We then applied *FineMAV* to genome-wide data from the 1000 Genomes Project (Phase 3) [37] to discover positive selection signals in Africa, East Asia, and Europe, and tested the results by examining whether or not: (1) our method was able to separate the other gold standard variants from the surrounding linked SNPs; (2) the gold standards as a group were found among the extreme outliers of the genome-wide distribution; and (3) *FineMAV* also enriched for genes identified in previous genome-wide selection scans with high *Selection Support Index* (*SSI*) values (Additional file 2).

**Table 1:** List of “gold standard” selected variants used for *FineMAV* calibration and validation

Gene	SNP	Population	Function
*ACKR1* ^a^	rs2814778	AFR	Malaria resistance [115–118]
*SLC39A4*	rs1871534	AFR	Zinc level [119]
*ABCC11*	rs17822931	EAS	Earwax and sweat type [120, 121]
*EDAR*	rs3827760	EAS	Hair shape and thickness [33, 122]
*HERC2*	rs12913832	EUR	Eye pigmentation [123–125]
*MCM6*	rs4988235	EUR	Lactose tolerance [35, 36]
*SLC24A5*	rs1426654	EUR	Skin pigmentation [34, 126]
*SLC45A2*	rs16891982	EUR	Skin pigmentation [126–128]

^a^Note that *ACKR1* is also known as *DARC* and the derived allele at rs2814778 is the Duffy O allele

*AFR* Africans, *EAS* East Asians, *EUR* Europeans

**Fig. 2:**
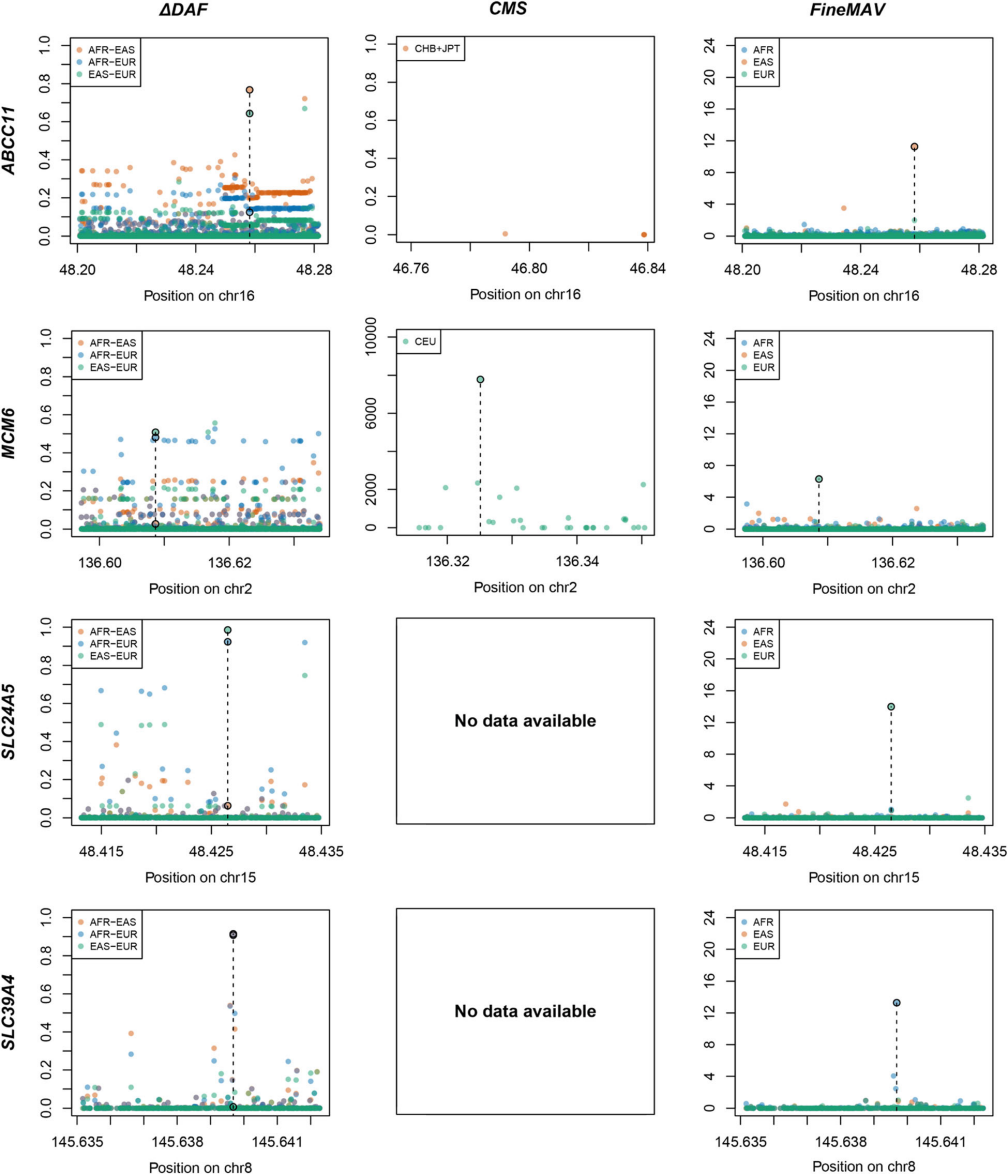
Comparison of *FineMAV* with existing approaches for pinpointing positively selected variants in the calibration set. *ΔDAF*, *CMS*, and *FineMAV* scores are shown for the genomic windows spanning genes from the gold standard calibration panel. *ΔDAF* and *FineMAV* were calculated from the 1000 Genomes Project Phase3 dataset [37] for Africans (AFR, *blue*), East Asians (EAS, *orange*), and Europeans (EUR, *green*). *CMS* scores for localized regions were downloaded from an online repository [39] and included: region8new and region152new calculated using the pilot phase of 1000 Genomes Project [129]. Variants with *CMS* value set to “nan” were not plotted; thus, some *CMS* plots are missing. Genomic positions are given in Mb according to GRCh37 for *ΔDAF* and *FineMAV*, and build NCBI36 for *CMS*. The selected variant is marked with a *dashed line. FineMAV* notably reduced the noise of neutral background variation, so that the selected variant is always the highest scoring one in any given gene. Note that the *y-axis* scale in the *CMS* plots is not standardized

**Fig. 3:**
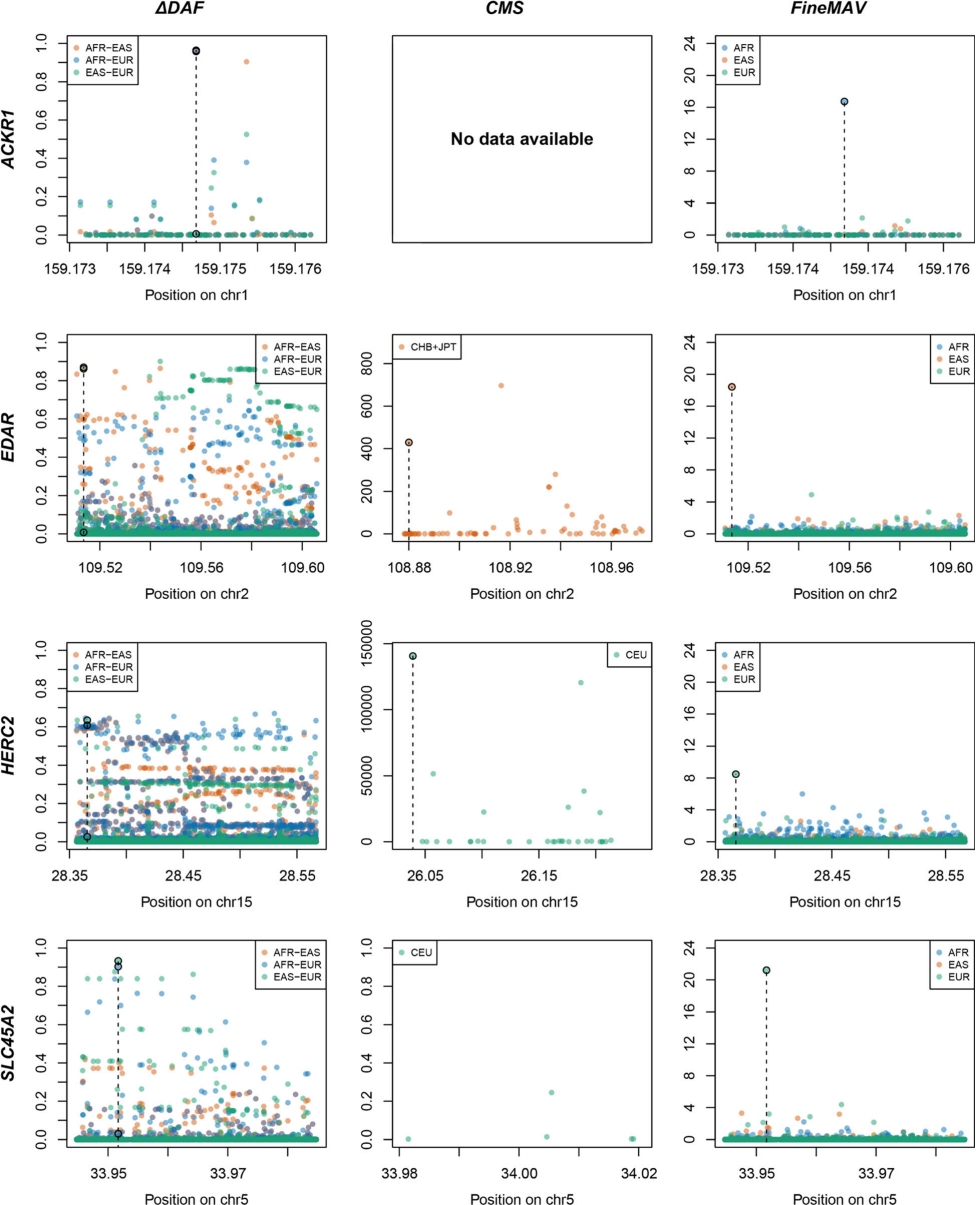
Comparison of *FineMAV* with existing approaches for pinpointing selected variants in the replication set. *ΔDAF*, *CMS*, and *FineMAV* scores are shown for the genomic windows spanning genes from the gold standard replication panel. *ΔDAF* and *FineMAV* were calculated from the 1000 Genomes Project Phase3 dataset [37] for Africans (AFR, *blue*), East Asians (EAS, *orange*), and Europeans (EUR, *green*). *CMS* scores for localized regions were downloaded from an online repository [39] and included: region34new, region104new, and SLC45A2old, all calculated using the pilot phase of 1000 Genomes Project [129]. Variants with *CMS* value set to “nan” were not plotted; thus, some *CMS* plots are missing. Genomic positions are given in Mb according to GRCh37 for *ΔDAF* and *FineMAV*, and build NCBI36 for *CMS*. The selected variant is marked with a *dashed line. FineMAV* notably reduced the noise of neutral background variation, so that the selected variant is always the highest scoring one in the given gene. Note that the *y-axis* scale in the *CMS* plots is not standardized

Results of the refinement of the signal of selection for the gold standard panel calibration and replication sets are shown in Figs. 2 and 3, respectively, together with the performance of methods relying on population-genetic data alone (*ΔDAF* – a standard measure of population differentiation [28] and *CMS* – a composite method [29, 39]). Our integrative approach successfully distinguished the positively selected variants from neutral background variation in all cases, whereas the standard methods were often unable to differentiate between the functional variant and its neutral proxies. Values of individual *FineMAV* components for each genomic window are shown in Additional file 1: Figure S2 and S3. Furthermore, we assessed how often the positively selected variant was the highest scoring one in a genomic window of 1000 SNPs in both the simulated and empirical data (1000 Genomes Project sequence data spanning the gold standard panel) according to three different tests (Table 2). In this comparison, we used two statistics relying on population genetic data alone (*ΔDAF* and *DAPxDAF* – population genetic component of *FineMAV*) and compared with our statistic *FineMAV* incorporating the measure of functionality. Inclusion of functionality improved the fine mapping of truly selected variants remarkably (Table 2). It is also worth noting that *DAPxDAF* is more sensitive to the signature of local adaptation than *ΔDAF* in the simulated data, especially for lower selection coefficients (Table 2 and Additional file 1: Figure S4 and S5).

**Table 2:** Different tests’ power to identify the selection driving SNP as the top scoring one in the genomic window of 1000 SNPs

Scenario	*ΔDAF*	*DAPxDAF*	*FineMAV*
Empirical data	0.75	0.75	1
Simulation s = 0.001	0	0	0.01
Simulation s = 0.007	0.23	0.44	0.75
Simulation s = 0.01	0.72	0.84	0.92

“Empirical data” means 1000 Genomes Project sequence data of the gold standard panel. “Simulation” is given for three different selection coefficients (s). "*DAPxDAF*" specifies *FineMAV* without functional prediction

**Fig. 4:**
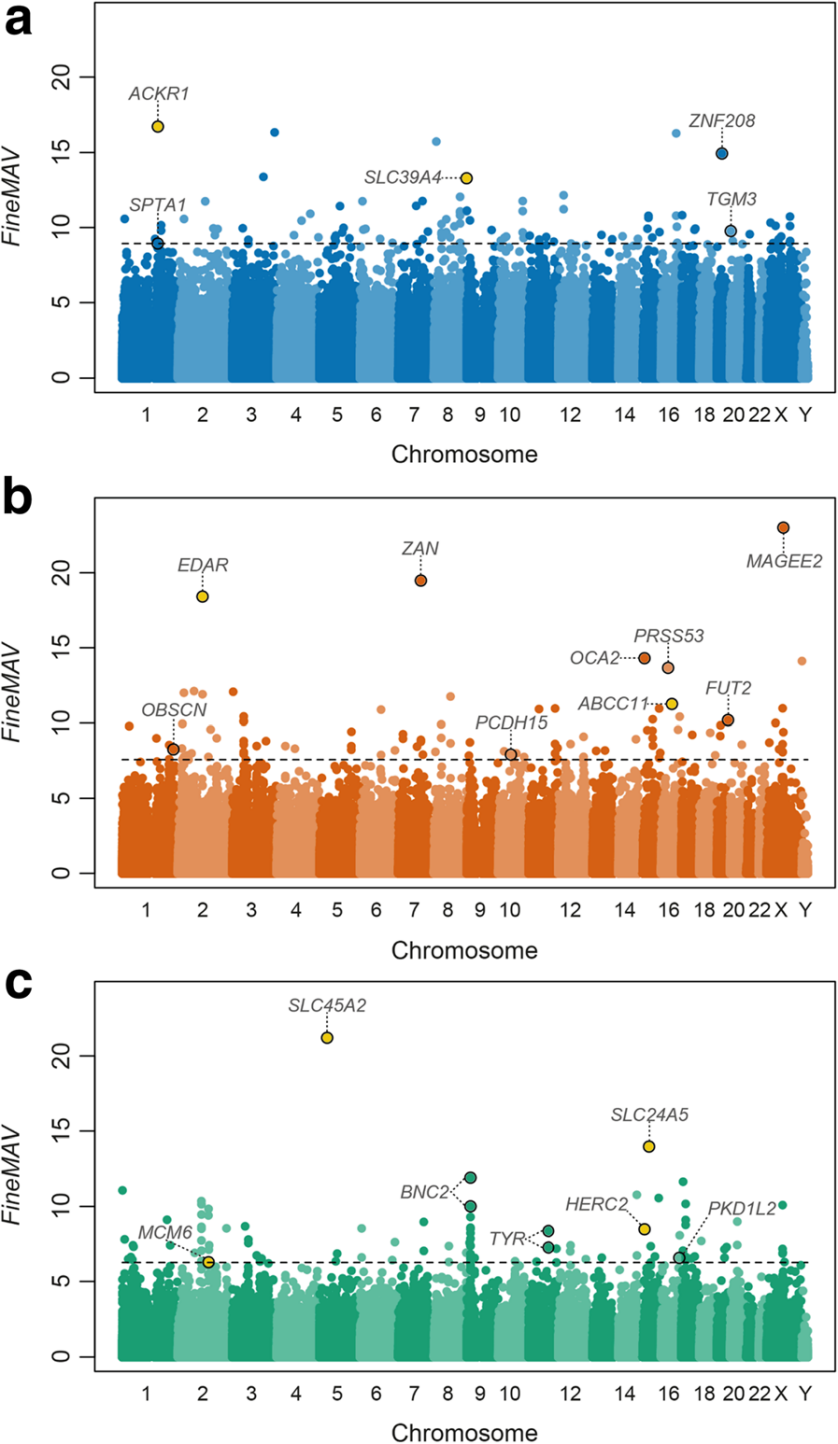
Manhattan plot of genome-wide *FineMAV* scores. *FineMAV* scores were calculated for genome-wide SNPs from 1000 Genomes Project Phase 3 [37] in three continental populations: (**a**) Africans (AFR, *blue*); (**b**) East Asians (EAS, *orange*); (**c**) Europeans (EUR, *green*). Each *dot* in the Manhattan plots represents a single SNP plotted according to coordinates in GRCh37. The threshold (*dashed lines*) was set to include the top 100 variants (top ~ 0.0004% of the whole-genome distribution). All gold-standard SNPs (*yellow dots* found among the top outliers) and other interesting candidate variants are labeled with the name of the gene they fall into

We then ranked all variants in the 1000 Genomes dataset according to their *FineMAV* value to identify extreme outliers in the upper tail of the empirical genome-wide distribution for each continent and examined whether or not the gold standard variants fell in the extreme tail. We indeed found all the gold standards to be high scoring (Fig. 4) (among the top 0.0004% of the whole-genome distribution [Additional file 1: Figure S6 and Additional file 3]) and set a conservative threshold to include the top 100 candidates per population (incorporating all gold standards and a total of 300 variants, out of more than 78 million derived alleles [Additional file 1: Figure S6 and Additional file 3]) for downstream analysis. Among those 300 *FineMAV* top-hits, we observed varying levels of allele frequency (*DAF* range of ~ 0.25–1) and allele sharing between populations (*DAP* range of ~ 0.38–1), all characterized by a functional *CADD* score prediction (in the range of ~ 11–47 with a mean of ~ 19). It is worth noting that although *FineMAV* prioritizes population-specific alleles, it also allows some degree of allele sharing between populations. The distribution of continental *DAF*, *DAP*, and *CADD* in the top *FineMAV* outliers in each population are shown in Additional file 1: Figure S7, S8, and S9, respectively.

### Functional validation in silico

To further evaluate our top *FineMAV* hits, we performed an in silico validation by searching the available literature for relevant functional information. *FineMAV*’s performance is supported by several lines of evidence. The first verification comes from the “gold standard” replication set (the best examples of validated causal adaptive variants). Not only did *FineMAV* replicate the signals in these well-known cases of strong election, but it also narrowed it down to the known single functional SNP, even in high LD regions. Positive controls extend to other variants that were not included in the “gold standard” panel, but whose prior evidence of causality is also strong, potentially providing additional support for our method. *FineMAV* rediscovered additional known SNPs implicated in eye, hair, and skin pigmentation in non-Africans, such as rs1800414 in *OCA2* (skin lightening in East Asians) [40–42], rs1042602 and rs1126809 in *TYR* (pigmentation and freckling in Europeans) [43–45], rs12350739 in *BNC2* (freckling and color saturation of human skin pigmentation in Europeans) [46], and also rs1047781 in *FUT2* (an enzyme-inactivating mutation conferring advantage in avoiding certain viral infections in East Asians) [11, 47], rs3211938 in *CD36* selected in Yoruba (protection against malaria and/or the metabolic syndrome) [48–50], and rs1229984 in *ADH1B* (protection against alcohol dependence in East Asians) [51–54].

Finally, *FineMAV* also identified a variant with no prior implication of functionality that was experimentally validated while our study was in progress, thus providing additional evidence of its performance. We picked up the missense SNP rs11150606 as the sixth top-scoring *FineMAV* variant in East Asians and noticed that it fell in *PRSS53*, whose function was then largely unknown. *PRSS53* encodes a polyserine protease called polyserase-3 (POL3S), which hydrolyzes peptide bonds. Subsequently, Adhikari et al. showed that *PRSS53* is highly expressed in the hair follicle and rs11150606 was associated with hair shape in East Asians [55]. The authors confirmed the functionality of rs11150606 by in vitro assays, showing that it affects processing and secretion of the gene product, with the derived allele contributing to the straight hair phenotype (similarly to the well-established gold standard *EDAR* variant) [55]. It can thus be considered another gold standard example demonstrating the validity of our method in picking up true functional selected variants.

Furthermore, we looked at the enrichment of genome-wide association study (GWAS) hits and expression quantitative trait loci (eQTLs) more generally among our top outliers (Additional file 2). GWAS enrichment was carried out in an LD framework (Additional file 2) instead of a simple overlap between *FineMAV* outliers and GWAS hits, because many GWAS studies are based on array-genotyping, rather than whole-genome sequencing data and are confounded by LD between functional and linked variants. We saw enrichment in GWAS hits among *FineMAV* outliers in Europeans and Eurasians (especially in the high LD category r^2^ ≥ 0.9) (Additional file 2). As the majority of GWAS studies were conducted in populations with European ancestry, the lack of enrichment in Africans and East Asians is not surprising. The directionality of the association was not available for all variants, but most of the annotated selected alleles (or their tagging SNPs) conferred protective effect, except a few derived alleles associated with increased risk of schizophrenia, type 2 diabetes, increased adiposity, lupus erythematosus, and degeneration of lumbar disc (Additional file 4). We also saw selection on alleles associated with increased height and lower age of puberty in Europeans [56], delayed eruption of permanent teeth in Eurasians [57], facial morphology in Africans, and chin dimples in Europeans and Asians [58] (Additional file 4).

Similarly, we saw an enrichment in eQTLs among *FineMAV* top outliers in Europeans and Eurasians as compared to a random expectation (*p* values of 0.04 and 0.03, respectively), although with lower significance than for GWAS signals. In a similar fashion, non-European ancestries are under-represented in eQTL databases. Over half of the top 100 *FineMAV* outliers in Europeans and Eurasians were annotated as significant eQTLs (Additional file 3).

### Novel candidate variants in Africa, East Asia, and Europe

Although we have thus far highlighted known variants replicated in our analysis, which serve as positive controls for evaluating our method’s performance, the vast majority of outliers discovered are novel and fall in non-coding regions (Additional file 1: Figure S10 and Additional file 2). We also identified variants on the X and Y chromosomes which have been under-represented in previous genomic scans [28, 29, 31, 39, 59–80], but further functional testing is needed to explore these findings. It is worth noting that the paucity of *FineMAV* hits on the Y chromosome (only one in the top 300) shows its strong dependence on the *CADD* score prediction.

We observed some high-scoring nonsense variants among our top candidates, suggesting pseudogenization of *PKD1L2* (an endogenous fatty acid synthase in skeletal muscle) [81] in Europeans, *ZNF208* (zinc finger and SRY-interacting protein) [82] in Africans, as well as *ZAN*, *OBSCN* (sacromeric signaling protein involved in myofibrillogenesis) [83] and *MAGEE2* (melanoma-associated antigen expressed in the brain) [84] in East Asians. *ZAN* is particularly interesting as it encodes a zonadhesin protein located in the acrosome that mediates the species specificity of sperm binding to the extracellular coat of the egg (zona pellucida) [85]. We find a signal of selection at a nonsense mutation (rs2293766) present at 51% frequency in East Asians, but virtually absent elsewhere.

*FineMAV* also highlighted rs6048066, a missense variant in *TGM3* in Africans. The *TGM3* gene product’s deficiency in humans has been linked to Uncombable Hair Syndrome, characterized by dry, frizzy, and wiry hair [86], while the *Tgm3* knockout mice exhibit rough-looking, curly, or brittle hair [87–89]. The missense variant we report here falls in the catalytic core of the protein, as does the mouse non-synonymous *we*^*Bkr*^ allele causing a wavy coat and curly whisker phenotype [89]. SNPs in *TGM3* have been weakly associated with hair diameter in humans [90] and proteomic profiling of human hair shafts identified TGase 3 as a major component of the hair fiber and revealed considerable variation among samples of different ethnic origins, with the lowest levels in African Americans and Kenyans [91]. We propose that this missense variant (rs6048066) might cause enzyme deficiency and contribute to African hair texture, hypothesized to have experienced strong positive selection in equatorial climates due to body-temperature regulation [92, 93].

Finally, regulatory variants are particularly interesting as they form the most abundant functional category among *FineMAV* outliers (Additional file 1: Figure S10 and Additional file 2) and are responsible for the bulk of human phenotypic variation [21, 35, 94]. However, the functional effect of regulatory variants remains difficult to predict and interpret. We find a signal of selection on rs12881545—an intronic regulatory variant falling in a promoter-flanking region and transcription factor binding site that scores as the top sixth variant selected in Europeans. The region surrounding rs12881545, although non-coding, is characterized by high conservation across taxa and the presence of DNaseI hypersensitivity. Our GWAS analyses revealed that rs12881545 is tagged by rs7141210 (r^2^ = 0.96) associated with lower age at menarche [56]. rs12881545 is also a direct eQTL and the selected allele increases *DLK1* expression (*p* value = 0.000015) [84]. DLK1 is an epidermal growth factor involved in differentiation of many tissues with strong links to adiposity and body growth. Furthermore, in accordance with the GWAS association [56, 95], aberrations in *DLK1* has been linked to central precocious puberty (a condition where puberty starts too soon in children) [96]. Although potentially pleiotropic, it could be that this regulatory variant modulates the expression level of *DLK1* and timing of menarche.

In-depth discussion of further novel alleles and speculation on the plausible selection pressures acting on them can be found in Additional file 2. The functional significance of these novel candidate variants presented here needs to be experimentally validated, but narrowing their signal of selection to a single most likely selected candidate SNP is a good starting point for such efforts.

### *FineMAV* analysis in Admixed Americans and South Asians

After the calibration of our method and an assessment of its performance in African, East Asian, and European populations, we applied it to the remaining 1000 Genomes Project populations: Admixed Americans (AMR) and South Asians (SAS). *FineMAV* revealed less population-specific selection in these populations (Additional file 1: Figure S11) due to population admixture (AMR) or shared ancestry (SAS). Nevertheless, a single strong outlier was observed in the SAS, found at 0.54 frequency there but virtually absent elsewhere: the missense SNP rs201075024 in *PRSS53* (Additional file 1: Figure S11.A). This is different from the non-synonymous variant in *PRSS53* in East Asians (previous section), but lies in close proximity, only 10 bp away (Additional file 1: Figure S12), which might indicate a similar functional consequence and convergent evolution of a hair-related phenotype, especially as both mutations are non-synonymous and have a similar molecular nature. Besides *PRSS53*, we see several potential signals of convergent or parallel evolution [1], i.e. selection on the same gene in geographically distant populations but on a different SNP (Additional file 3); however, only in the case of *PRSS53* do the similarities in locations and consequences of the SNPs provide a strong priori likelihood of convergent evolution.

In the AMR, even though admixture decreases the *FineMAV* signal, the gene flow into the Americas affects the frequency of derived Native American alleles, but not their purity (as private American alleles would still be found exclusively in Americas giving high *DAP* values). In the case of common derived alleles selected to high frequencies before the admixture event, a *FineMAV* signal should still be detectable (assuming their high functional prediction) in the extreme tail of the whole-genome distribution; the top three scores were missense variants: rs148608573 in *MAP7D1*, rs142326775 in *ZNF438*, and rs34890031 in *LRGUK* (where the mouse homologue is essential for multiple aspects of sperm assembly and function) [97] (Additional file 1: Figure S11.B).

## Discussion

The aim of this study was not to perform another selection scan and it should not be interpreted in that way. Instead, it aims to refine a proportion of local adaptations to a single variant and prioritize positively selected candidates for further functional validation, as existing methods often do not pinpoint the selected SNPs. Furthermore, this paper does not focus on experimental follow-up of novel selection signals, but rather provides a decision-making algorithm for identifying high-priority causal variants for subsequent experimental work. To achieve these aims, we introduced the *FineMAV* statistic which combines measures of population differentiation, derived allele frequency, and molecular functionality. Incorporation of diverse functional annotations (such as predictors of deleteriousness) should improve the pinpointing of likely selected variants and lower FDR, as it has in the detection of disease-causing variants [98]. It is worth noting that variants classified as damaging alter the level or biochemical function of a gene product, but do not necessarily decrease the reproductive fitness of carriers [38, 99]. The functional consequence of the “damaging” change for a person depends on many factors and can be either negative or positive (as, for example, deficiency alleles might be either beneficial or detrimental) depending on the environmental context. For instance, variants disadvantageous in one environment can be favored under different conditions, e.g. *CPT1A* [16, 17].

*FineMAV* was calibrated and tested using a gold standard panel of the eight best examples of experimentally validated functional variants underlying signals of positive selection in humans and was able to identify the known functional candidate in all instances (Figs. 2 and 3). Using the 1000 Genomes Project Phase 3 dataset [37], we then ranked all genome-wide SNPs based on their *FineMAV* value and identified extreme outliers in the upper tail of the empirical genome-wide distribution in Africa, Europe, and East Asia (Additional file 3). *FineMAV* rediscovered many known variants with prior evidence for being causal of positive selection signals, which were not part of the calibration set, providing additional support for our method. We also identified potential functional variants in other genes reported to be under strong positive selection in the literature (with strong *SSI* scores; Additional file 5) where the specific positively selected variant had not been confirmed, including *LPP*, *PCDH15*, and *PRSS53*. The selection signal in *PCDH15* and *PRSS53* was attributed to a single missense variant per population (rs4935502, rs11150606, and rs201075024, respectively), replicating and extending the results obtained by *CMS* [39, 55].

The signal in *BNC2* was particularly strong in Europeans, as reflected by a cluster of 12 SNPs found among the top 100 hits in the *FineMAV* distribution (Fig. 4c). The hypothesized functional SNP (the intergenic rs12350739) was the second highest-scoring *BNC2* variant in our analysis and has been reported to be a functional eQTL as it falls in a highly conserved melanocyte-specific enhancer and regulates *BNC2* transcription [46]. The highest-scoring *BCN2* variant (rs10962600) might also contribute to the differential expression of *BNC2* isoforms as several regions inside and outside of the *BNC2* gene contain enhancer features [46]. Interestingly, *BNC2* has been highlighted as present in a region of the human genome that shows Neanderthal ancestry (Additional file 1: Figure S13), suggesting that Neanderthal introgression might have provided modern humans with adaptive variation for skin phenotypes involving BNC2 [46, 100–102]. Furthermore, a cluster of high-scoring SNPs in *FineMAV* analysis might more generally be indicative of introgression as a source of adaptive variation, as opposed to advantageous de novo mutations that usually arise individually. Although we cannot exclude the possibility of more than one causal SNP in regions introgressed from archaic hominins (especially those falling in regulatory elements), it seems that *FineMAV* may have low resolution in cases of adaptive introgression. We also found other *FineMAV* outliers in regions proposed to be adaptively introgressed from an archaic source (27 SNPs in total) in *GNAI2*, *GPATCH1*, *IRF6*, *POU2F3*, *RASSF1*, *SEMA3F*, and *SLC38A3* (Additional file 1: Figure S13) [100–103], suggesting that some of the candidates might be of archaic rather than de novo origin. However, the origin of the adaptive mutations is not the focus of this study and has been considered elsewhere [100–103]. Apart from *BNC2*, several other introgressed SNPs also showed GWAS associations, including *IRF6* (cleft lip), *GPATCH1* (bone density), and, most interestingly, a high-LD eQTL region on chromosome 3 spanning *GNAI2*, *HYAL1*, *HYAL2*, *RASSF1*, *SEMA3F*, and *SLC38A3* in East Asians associated with keloid scar formation resulting from dysfunction of the wound-healing processes [104]. It has been shown that keloid susceptibility varies across ethnicities with higher incidence in Africans and East Asians, and darker-skinned populations in general [105, 106].

Finally, *FineMAV* picked up variants with modest to high derived allele frequency in the range of ~ 0.25–1 within continental populations (Additional file 1: Figure S7). Most classical methods detect only extreme allele frequency differences between populations, which are less likely to arise by chance [22]. On the other hand, highly functional alleles are less likely to be subjected to random changes in their frequency; thus, it seems that filtering out neutral variation by applying functional information might allow more examples of weaker sweeps (potentially including selection on standing variation) to be discovered, which are characterized by more modest allele frequency shifts [4, 22], although our method has no power to detect low selection coefficients that do not produce population differentiation patterns.

Functional validation of candidate signals of selection is a current roadblock in the field, limiting both our understanding of the modes and importance of positive selection and the independent evaluation of methods to detect it. Modeling of non-pathological human genetic variation in cell or animal systems, however, has received only limited attention to date [107]. Our study misses some genuine selected variants, but our prioritization aims to enrich for true positives, which is what matters for studies that may spend years examining individual candidates in cellular or animal models. For example, the reason for selection of the *TRPV6* haplotype containing three derived non-synonymous substitutions observed in non-African populations remains enigmatic despite detailed functional characterization of selected and non-selected forms at the cellular level [108]. Although it remains possible that the ancestral and derived forms differ in aspects that were not tested or can only be observed at the whole-organism level [108], none of the three candidate sites was supported by our *FineMAV* analysis (in both selection scenarios *n* = 3 [AFR, EAS, EUR] and *n* = 2 [AFR, EAS + EUR]; see “Methods”) since their score was low (*FineMAV* ~ 1 for each variant in the EAS + EUR scenario). Therefore, we see them as weak candidates for causality and would not suggest a high priority for modeling them.

## Conclusions

Modeling human selection in cell or animal systems is challenging since relevant phenotypic consequences (often very subtle) might be overlooked. Some phenotypes may be manifest only in certain conditions, such as the presence of specific pathogens or environmental stresses, and might be missed even by association studies in humans [7, 33]. The inability to directly demonstrate phenotypic consequences in a limited set-up, therefore, does not entirely rule out the possibility that a variant has been selected [21]. Nonetheless, regardless of challenges like these, cell and animal models often provide the best way to test hypotheses regarding recent human evolution [33]. *FineMAV* now offers an improved way to identify specific variants for these tests and paves the way for systematic identification of selected alleles driving phenotypic differences among human populations, future functional studies of individual loci, and more general understanding of the circumstances in which local adaptations occur.

## Methods

### Fine-mapping of adaptive variation

Fine-Mapping of Adaptive Variation (*FineMAV*) is designed to refine a signal of selection to a single most likely selected variant and thus to differentiate it from the passenger variants for functional follow-up studies. *FineMAV* is most relevant for targets of recent or ongoing local positive selection underlying local adaptations in humans following the out-of-Africa migration (within the last ~ 60,000 years) and can be applied to a region of prior interest, or to the whole genome, for discovering novel positively selected variants. It could also potentially address old selection in the human lineage (preceding the out-of-Africa expansion; see “*FineMAV* calibration” below), but this is not the main focus of this study.

A *FineMAV* score was calculated for the derived allele of each SNP by combining its *DAP*, *DAF*, and functional prediction score (the *CADD* PHRED-scaled C-score) [38] (Eq. 1). The rationale behind doing so is that variants predicted to be non-functional are (within the limitations of the prediction) likely to be neutral, since natural selection can only act directly on variants that confer a phenotypic effect. If an allele is predicted to be highly functional and rare, it will often be deleterious; but it cannot be harmful if it is both functional and common, and may potentially be adaptive. Importantly, all three metrics are allele-specific (rather than site- or gene-specific) and consequently allow direct evaluation of individual alleles. We simply scaled and combined the metrics to obtain a single measure giving high values to derived alleles that are common, population-specific, and functional (Eq. 1). Individual components are introduced in the following sections. Although *FineMAV* can be also applied to ancestral alleles by calculating their allele frequency and purity, detection of selection on segregating ancestral alleles would be limited by extensive sharing of ancestral alleles worldwide (across different populations) and their low purity scores. Therefore, it is unlikely to detect selection on segregating ancestral alleles that do not produce a high population differentiation signature.

Equation 1. Fine-Mapping of Adaptive Variation. To compute *FineMAV* per derived allele across *n* populations, suppose *i* ∈ {1, 2, …, *n*} and let *DAF*_*i*_ be derived allele frequency in population *i*.

$$ FineMA{V}_i= DAP\times DA{F}_i\times CADD $$

#### Measure of population differentiation

We used an allele frequency differentiation method as a signature of local selection in *FineMAV*. We chose a measure of population structure differing somewhat from existing methods, as it: (1) operates at the variant level; (2) does not rely on the hard sweep assumptions of strong LD and SFS signatures (which can be erased by recombination); (3) is sensitive to many types of selection including classic sweeps and selection from standing variation; and (4) detects recent human adaptations [4, 21, 22, 24, 25]. Alternative methods based on extended LD or distortion of SFS are characterized by low genomic resolution (summary statistics are calculated for large genomic windows) [109] and do not allow single candidate variants to be pinpointed, which is the key aim of this manuscript. Furthermore, such methods lead to signals mainly in cases of strong hard sweeps (known to be rare in human evolution). Therefore, such tests were not incorporated into the *FineMAV* score.

Any selection event, regardless of its mode, will eventually produce an excess of allele frequency differentiation between populations as long as: (1) it has taken place in one population but not in another, and the allele was at low frequency when first favored; (2) there is variation in selection coefficient over space; (3) migration and gene flow between the populations have been restricted; and (4) there has been enough time for selection to act [4, 22]. Even if an allele is equally advantageous in all environments, but its selection happened in a regionally restricted manner, the selected variant will be concentrated around its geographic origin due to limited dispersal [4, 7].

We proposed and applied a new measure of population differentiation called *DAP. DAP* is related to *ΔDAF* [28] and other pairwise comparison-based methods, but is able to summarize population differentiation (spatial pattern of the derived allele) across many populations in a single measure for each variant. *DAP* is a measure of derived allele entropy based on Gini impurity [110] and describes how unequally the derived allele is distributed among diverse populations. *DAP* operates on derived allele counts in a population sample when distinct groups are equally represented and is calculated according to Eq. 2. When population groups are not equally represented, derived allele count can be estimated from derived allele frequency. *DAP* counts derived allele occurrences across populations and describes their spatial distribution, reaching its maximum of 1 when all cases (derived alleles) fall into a single population category and penalizes allele sharing between different populations. The magnitude of the penalty can be controlled by the *x* parameter (“penalty parameter”) depending on the user’s purposes and the number of populations being compared (*n*) (see “*FineMAV* calibration”). *x* is an inherent parameter of the population differentiation test, as without it *DAP* would not measure entropy and would remain constant (equal to 1) for all alleles. We calibrated *x* using a subset of our gold standards (see “*FineMAV* calibration”). It is worth noting that *DAP* is a measure of derived allele purity (or “privateness”) and scores highly for both rare and common alleles found exclusively in a single population (characterized by high population differentiation) and therefore needs to be combined with a measure of allele abundance (*DAF*) in order to detect local adaptation.

Equation 2. Derived allele purity. To compute derived allele purity per site (*DAP*) across *n* equally represented populations, suppose *i* ∈ {1, 2, …, *n*} and let *d*_*i*_ be derived allele count in population *i*.

$$ {d}_N=\sum \limits_{i=1}^n{d}_i $$

$$ {f}_i=\frac{d_i}{d_N} $$

$$ DAP=\sum \limits_{i=1}^n{f}_i^x $$

#### Measure of allele prevalence

We estimated allele abundance using two alternative approaches: (1) global derived allele frequency; and (2) continental derived allele frequency. In both cases, *DAF* is in the range of 0–1. We obtained the continental *DAF* by averaging *DAF* across all populations within each continent and calculated global *DAF* for each variant by averaging continental *DAF*s. Both approaches yielded similar results (almost identical lists of top 100 extreme outliers). The main difference between these two measures of allele prevalence is that incorporation of global *DAF* results in a single *FineMAV* score for each derived allele (which is then assigned to a single population based on the difference in derived allele frequency between examined populations), while application of continental *DAF* leads to calculation of *FineMAV* scores for each population separately. Global *DAF* is *n*-dependent, while continental *DAF* remains constant regardless of *n*, thereby making *FineMAV* values comparable across different values of *n*. Here, we report results incorporating continental *DAF*. The combined measure of *DAPxDAF* is the population genetic component of the *FineMAV* test that detects the signature of local adaptation.

#### Measure of functionality

It is crucial that variant-level functional inferences are based on whole-genome measures to ensure that all potentially selected variants are treated equally. We needed a measure of functionality to be allele-specific and applicable to all variation, both coding and non-coding, since many signals of selection localize in regulatory elements or intergenic regions [21, 29]. As proteins are usually involved in multiple processes through complicated interaction pathways with other proteins, amino acid change in one protein may affect diverse traits, i.e. pleiotropic phenotypes [33]. In general, pleiotropic changes are thought to be disadvantageous [94], so it is believed that a great deal of human phenotypic variation is based in regulatory variation [21, 35, 94]. Thus, different sets of annotations for coding and non-coding variation would make it challenging to compare these distinct variant categories and consensus methods combining multiple annotations, each with its own strengths and weaknesses, are especially needed here for unbiased prioritization of variants [38]. In our analysis, we used the *CADD* (v1.2 PHRED-scaled C-score), which integrates 63 diverse genome annotations into a single measure for each variant and in theory can take a value in the range of 0–99 [38].

#### *FineMAV* calibration

We compiled a gold standard panel of the eight best examples of experimentally validated functional variants underlying signals of positive selection which are linked to specific phenotypic consequences (Table 1) and calibrated *FineMAV* using population-scale sequence data (1000 Genomes Project Phase 3) of genomic windows spanning half of the gold standards (randomly chosen from each population). We then examined if the calibration results are sensitive to different combinations of gold standards in a quantitative and reproducible manner.

Increment of the penalty for allele sharing (*x*) increases the difference between the *FineMAV* score of the differentiated selected SNPs and less differentiated nearby neutral variants. The magnitude of this difference increases with increasing *x*, reaches a plateau, and then decreases for larger values of *x*. For each gold standard gene (empirical data), we calculated the fold change between the *FineMAV* scores of the selected variant and the highest scoring neutral background SNP using different values of *x* (1 ≤ *x* ≤ 4). We then selected the optimal values of *x* for each gold standard gene that maximizes the difference between the selected and neutral variants (Additional file 1: Figure S14). Based on our calibration set, we decided to set the penalty parameter *x* to a consensus value of 3.5.

If our calibration analysis relied on a single gold standard gene, the optimal *x* would fall in the rage of 2.5 ≤ *x* ≤ 4 (Additional file 1: Figure S14). The same holds true for different combinations of gold standards. Although some gold standards reached a plateau earlier than others, and some of them did not reach a plateau in the examined interval at all, it seems that the minimal value of 2.5 is large enough to differentiate between selected and neutral SNPs in all cases (Additional file 1: Figure S14). Although further increase of *x* (>4) improves the fold change between some of the gold standards and neutral variants, we also noticed a decrease in the fold change in the examined range of *x* and decided not to extend this range in our calibration analyses.

Furthermore, we examined the overall rank of gold standards in the empirical whole-genome distributions of *FineMAV* and *DAPxDAF*. The average rank improves dramatically with increasing *x* until 2.5, and then plateaus (with further decrease above 4 in case of *DAPxDAF* distribution) (Additional file 1: Figure S15). The optimal value of *x* for highest ranks is seen at *x* = 3.5 and 4 (all gold standards among top 100 genome-wide outliers). Similarly, *FineMAV*’s and *DAPxDAF*’s power to detect the selection-driving SNP as the highest scoring in a genomic window of 1000 nearby SNPs does not increase substantially with *x* > 2 (using both simulated and empirical data; Tables 3 and 4). Finally, we performed overlap analyses of the top 100 *FineMAV* outliers from the empirical whole-genome distribution across different values of *x* (from 1 to 4) (Additional file 1: Figure S16). We conclude that a value of *x* from ~ 3 to 4 is optimal and further increment does not improve *FineMAV* analyses usefully (very similar set of top 100 outliers) (Additional file 1: Figure S16). We recommend *x* = 3.5 (in three populations comparison; *n* = 3) as an optimal penalty in *FineMAV* analyses, although higher penalties would yield very similar results. Note that this calibration was carried out using gold standards across three continental populations (*n* = 3) and *x* is sensitive to *n*. To see that: for maximally differentiated derived alleles (observed in one population only) *DAP* is constant (*DAP*_*max*_ = 1) and insensitive to n, while at the other extreme, minimally differentiated derived alleles (with the same frequency in all populations), *DAP* depends on *n* and *DAP*_*i*_ > *DAP*_*i*+1_ (1 < *i* ≤ *n*). To adjust for this and keep the *FineMAV* value insensitive to *n*, the *x* parameter for lower *n* values needs to be higher. Additional file 1: Figure S17 specifies the values of *x* for different values of *n* that make *FineMAV* values comparable across different values of *n*.

**Table 3:** *FineMAV*’s power to identify the selection-driving SNP as the top scoring one in a genomic window of 1000 SNPs across different values of *x* parameter

Scenario	*x* = 1	*x* = 1.5	*x* = 2	*x* = 2.5	*x* = 3	*x* = 3.5	*x* = 4
Empirical data	0.5	0.88	1	1	1	1	1
Simulation s = 0.007	0.6	0.7	0.71	0.72	0.74	0.75	0.76
Simulation s = 0.01	0.82	0.88	0.9	0.92	0.92	0.92	0.92

“Empirical data” means 1000 Genomes Project sequence data of the gold standard panel. “Simulation” is given for two different selection coefficients (s)

**Table 4:** *DAPxDAF*’s power to identify the selection-driving SNP as the top scoring one in a genomic window of 1000 SNPs across different values of *x* parameter

Scenario	*x* = 1	*x* = 1.5	*x* = 2	*x* = 2.5	*x* = 3	*x* = 3.5	*x* = 4
Empirical data	0	0.75	1	1	1	1	1
Simulation s = 0.007	0.08	0.39	0.44	0.43	0.44	0.44	0.45
Simulation s = 0.01	0.41	0.84	0.84	0.84	0.84	0.84	0.85

“Empirical data” means 1000 Genomes Project sequence data of the gold standard panel. “Simulation” is given for two different selection coefficients (s)

This calibration is robust to different combinations and number of gold standards used in the analysis and is supported by both empirical and simulated data. We encourage modified values of *x* if users wish to apply *FineMAV* to different species characterized by different levels of population differentiation or to different modes of selection, following the calibration framework presented here.

#### Balance between differentiation and functionality

The penalty parameter *x* also tunes *DAP* in relation to *DAF* and *CADD* and controls the balance between population differentiation and the prediction of functionality. The magnitude of the penalty for allele sharing in our population differentiation test (*DAPxDAF*) needs to fit the purpose of detecting selected alleles. We aim to pick up highly functional variants among the most differentiated variants (not the other way around) with the minimal cut-off for functionality at ~ 10 (variants with *CADD* scores below this threshold are considered non-functional).

When the *x* parameter is set to 1, population differentiation is not taken into account (*DAP* is constant and equals 1 for all variants) and *FineMAV* (or rather *DAFxCADD*) picks up derived alleles of high frequencies (often nearly fixed in the human lineage) with a strong prediction of functionality that are not differentiated between populations (e.g. the stop mutation in *CASP12* - rs497116) [9] (green tail in Additional file 1: Figure S18, S19 and S20; *x* = 1). We provide a list of such variants in Additional file 6. 77 of the top 100 such outliers were shared between Africans, East Asians, and Europeans. They could represent old selection events in the human lineage (presumably preceding the out-of-Africa expansion) and are potentially interesting, although beyond the scope of this study which focuses on recent selection and population diversification.

In the calibration stage, we needed to find the value of the *x* penalty parameter that assigns low scores to the background variation and highly functional derived alleles nearly fixed on the human lineage in the window around the selected variant. Imagine two scenarios. In scenario 1: a maximally differentiated derived allele that is exclusively fixed in population *i* but absent elsewhere (*DAP*_*max*_ = 1), which implies a maximal frequency (*DAF*_*i*_ = 1), and is predicted to be functional (*CADD* = 20). In this scenario, *FineMAV* = 20 and would be constant regardless of *n* (number of populations used in the analysis). Alternatively, in scenario 2, for a derived mutation that is fixed in all populations (*DAF*_*i*_ = 1) and is highly functional (*CADD* = 45) we need to penalize for allele sharing between populations to keep *DAP* (and consequently the *FineMAV* value) at a low level relative to scenario 1. *x* set to ~ 3 (and above) fits all above criteria (Additional file 1: Figures S18–S20). Increment of *x* removes the tail of nearly fixed derived alleles of high *CADD* prediction which disappears around *x* = 3 and 3.5 and leaves the most differentiated variants that are predicted to be functional (with *CADD* scores > 10) (Additional file 1: Figures S18–S20). Further increase of *x* (within the interval examined) has little effect on the results (see “*FineMAV* calibration” above and Additional file 1: Figures S18–S20). The penalty parameter *x* set according to Additional file 1: Figure S17 (3.5 and > 3 population comparison) is sufficient to give low scores to highly functional nearly fixed alleles (scenario 2: *DAP* ~ 0.064 and *FineMAV* ~ 2.88, which is at least seven times lower than the gold standard calibration set).

#### *FineMAV* calculation in 1000 Genomes Project samples

*DAF* and *DAP* values were calculated from the 1000 Genomes Project Phase 3 data release [37] using a custom script; *CADD* PHRED-scaled C-scores v1.2 were obtained from an online repository [38]. We ran our analysis for both autosomes and sex chromosomes, focusing initially on three continental populations: Africans (AFR), East Asians (EAS), and Europeans (EUR). We ran it in two contexts: (1) to re-discover continent-specific positive selection signals in Africa, East Asia, and Europe (*n* = 3; *x* = 3.5); and (2) to analyze selection that happened outside of Africa by pooling East Asians and Europeans together (*n* = 2; *x* = 4.96). Although our study focuses on local adaptation driving population differentiation at the continental scale, *FineMAV* can be also applied to study signals of selection within continents. It is also possible to investigate signals of selection shared between populations by relevant population grouping depending on the user’s purposes, e.g. selection outside Africa by pooling East Asians and Europeans together (Additional file 3).

*FineMAV* was calculated for derived alleles (annotated according to Ensembl) [111, 112] using a custom script (SNPs only). We applied a conservative *FineMAV* cut-off to include only the top 100 candidate variants in each continental population (which incorporated all gold standards and gave a total of 300 variants corresponding to the top ~ 0.0004% of the whole-genome distribution) for our downstream enrichment analysis (Additional file 2).

Subsequently, we also ran *FineMAV* in AMR and SAS from the 1000 Genomes Project Phase 3 data release [37], together with the three main continental populations, as follows: AFR, AMR, EAS, EUR; *n* = 4; *x* = 2.98 and AFR, EAS, EUR, SAS; *n* = 4; *x* = 2.98, to investigate population-specific local adaptation in those populations.

### Simulation analyses

Simulations assessing *FineMAV*’s performance were limited by the unknown relationship between the prediction of functionality (*CADD* score) and the selection coefficient. Although the functional range of *CADD* scores has been estimated, its FDR and sensitivity are poorly understood, while *FineMAV*’s performance is closely tied to the accuracy of the functional annotation. Nevertheless, we performed simulation analyses using individual-based forward-time simulations implemented in simuPOP v1.1.7 [113] to assess the power (true positive rate) and FDR of the *FineMAV* algorithm. We simulated three populations with a set of demographic parameters (starting effective population size, migration rate, and time of divergence) similar to estimates in European, African, and East Asian populations accordingly to published values [114]. We simulated a genomic window of 1000 SNPs with one SNP per window under selection in one population. The probability of recombination between two SNPs was set to increase with increasing physical distance between sites. The starting derived allele frequency for the selected marker was set to 0.01, while the allele frequencies of the remaining neutral SNPs were drawn from a beta distribution. Each SNP was assigned a *CADD* score value as follows:

1. Neutral SNPs were randomly assigned a *CADD* score value drawn from the genome-wide *CADD* distribution of derived alleles seen at ≥ 2% frequency in the 1000 Genomes Project Phase 3. Our simulation does not include purifying selection against pathogenic variants with high *CADD* values, so the derived allele frequency cutoff was set to 2% (approximately the minimal frequency at which a neutral derived allele should be seen at least once in a homozygous state in a population of the Phase 3 size) to remove rare deleterious variants from the *CADD* distribution.
2. We assumed that the *CADD* score distribution of selected variants is high and corresponds to known functional variants (which is supported by the *CADD* predictions of the gold standard panel). Based on this assumption, the *CADD* score for the selected SNP was drawn from the outlier distribution in the range of 10.78–47 (see below and “Results” section).

We then simulated four scenarios under the additive selection model with different selection coefficients: s = 0.001, s = 0.007, s = 0.01, and s = 0 (no selection); and a sample size of 500 individuals in each population. The populations were sampled after 1000 generations of selection and drift. Each scenario was replicated 100 times. The *FineMAV* algorithm was subsequently applied to each output dataset. We then checked how often the selected variants fall outside of the neutral *FineMAV* distribution. To determine the upper end of the neutral distribution we bootstrapped 1000 *FineMAV* values from the simulated neutral variation 100 times and took the maximum sampled value as our cut-off (set to *FineMAV* of 10.7).

## Additional files

Additional file 1: Supplementary figures [130, 131]. (PDF 16467 kb)

Additional file 2: Description of meta-analysis, enrichment analyses, and novel candidates found in this study. (DOCX 182 kb)

Additional file 3: Top 100 *FineMAV* SNPs in each continental population (*x* = 3.5). (XLSX 92 kb)

Additional file 4: GWAS hits among the top 100 *FineMAV* SNPs within each continental population. GWAS_SNP column specifies GWAS hits linked to given *FineMAV* SNPs. r^2^ column specifies the level of LD between the *FineMAV* SNP and the closest GWAS SNP. Genomic positions and the direction of genome-wide association are given for *FineMAV* SNPs. DER, derived allele of *FineMAV* SNP; ANC, ancestral allele of *FineMAV* SNP. Grayed variants might underline the same selection event. (XLSX 59 kb)

Additional file 5: Top *SSI* protein-coding genes. (XLSX 60 kb)

Additional file 6: Top 100 *FineMAV* SNPs in each continental population (*x* = 1). (XLSX 77 kb)

## Abbreviations

AFR: Africans
AMR: Admixed Americans
*CADD*: Combined annotation-dependent depletion
*CMS*: Composite of multiple signals
*DAF*: Derived allele frequency
*DAP*: Derived allele purity
EAS: East Asians
eQTL: Expression quantitative trait loci
EUR: Europeans
FDR: False discovery rate
*FineMAV*: Fine-mapping of adaptive variation
*F*_*ST*_: Wright’s fixation index
GTEx: Genotype-Tissue Expression project
LD: Linkage disequilibrium
s: Selection coefficient
SAS: South Asians
SFS: Site frequency spectrum
SNP: Single nucleotide polymorphism
*SSI*: Selection support index
*ΔDAF*: Difference in derived allele frequency
