# Supplementary material for: FineMAV: prioritizing candidate genetic variants driving local adaptations in human populations
Source: Genome Biol. 2018 Jan 17;19:5. doi: 10.1186/s13059-017-1380-2 (PMC5771147; doi:10.1186/s13059-017-1380-2)
Supplement: Supplementary file 1 — Supplementary figures [130, 131]. (PDF 16467 kb) [file 13059_2017_1380_MOESM1_ESM.pdf]

FineMAV: Prioritizing candidate genetic variants driving local adaptations in human populations

Michal Szpak,[1]* Massimo Mezzavilla,[1,2] Qasim Ayub,[1,3] Yuan Chen,[1] Yali Xue,[1] Chris Tyler-Smith[1]**

[1] Wellcome Trust Sanger Institute, Wellcome Genome Campus, Hinxton CB10 1SA, UK.

[2] Division of Experimental Genetics, Sidra Medical and Research Center, Doha, Qatar.

[3] Present Address: School of Science, Monash University Malaysia, Bandar Sunway, Selangor Darul Ehsan, Malaysia.

* ms30@sanger.ac.uk

** cts@sanger.ac.uk

Additional file 1

Figure S1. Simulated distribution of *FineMAV* scores for variants under selection. Three selection scenarios of varying selection strength were plotted: s=0.001, s=0.007 and s=0.01. Distributions of *FineMAV* values for selected variants from each scenario are shown as box-plots. The red dotted line represents the upper end of the neutral distribution. TPR and FDR indicate the true positive and false discovery rates.

Figure S2. *FineMAV*'s components for 'gold standard' genes in the calibration set. *DAP*, *DAF* and *CADD* scores are shown for the genomic windows spanning genes from the gold standard calibration panel (all calculated from the 1000 Genomes Project Phase3 dataset [37]). *DAF* indicates continental derived allele frequency for Africans (blue), East Asians (orange) and Europeans (green). Genomic positions are given in base pair (bp) according to GRCh37. The selected variant is marked with a dashed line.

Figure S3. *FineMAV*'s components for 'gold standard' genes in the replication set. *DAP*, *DAF* and *CADD* scores are shown for the genomic windows spanning genes from the gold standard calibration panel (all calculated from the 1000 Genomes Project Phase3 dataset [37]). *DAF* indicates continental derived allele frequency for Africans (blue), East Asians (orange) and Europeans (green). Genomic positions are given in bp according to GRCh37. The selected variant is marked with a dashed line.

Figure S4. Comparison of *ΔDAF*, *DAPxDAF* and *FineMAV* for pinpointing positively-selected variants in the calibration set.

ΔDAF, *DAPxDAF* and *FineMAV* scores are shown for the genomic windows spanning genes from the gold standard calibration panel; all calculated from the 1000 Genomes Project Phase3 dataset [37] for Africans (AFR, blue), East Asians (EAS, orange) and Europeans (EUR, green). Genomic positions are given in bp according to GRCh37. The selected variant is marked with a dashed line.

Figure S5. Comparison of *ΔDAF*, *DAPxDAF* and *FineMAV* for pinpointing positively-selected variants in the replication set.

ΔDAF, *DAPxDAF* and *FineMAV* scores are shown for the genomic windows spanning genes from the gold standard replication panel; all calculated from the 1000 Genomes Project Phase3 dataset [37] for Africans (AFR, blue), East Asians (EAS, orange) and Europeans (EUR, green). Genomic positions are given in bp according to GRCh37. The selected variant is marked with a dashed line.

Figure S6. Genome-wide distribution of *FineMAV* scores.

Genome-wide distribution of *FineMAV* scores in each continental population are shown on the left. The right panel shows *FineMAV* score distribution of variants matching continental derived allele frequency of our top outliers ($DAF \geq 0.24$). Dashed vertical lines indicate *FineMAV* cutoffs to include the top 100 variants in each population. Even after accounting for *DAF*, *FineMAV* identifies extreme outliers.

Figure S7. Derived allele frequency distribution among the top 100 *FineMAV* SNPs within each continental population.

Figure S8. Derived allele purity distribution among the top 100 *FineMAV* SNPs within each continental population.

Figure S9. CADD score distribution among the top 100 *FineMAV* SNPs within each continental population.

Figure S10. Functional consequences of *FineMAV* top outliers as compared to random expectation.
The 100 top outliers from each continental population (AFR, EAS, EUR) were pooled together. The chart uses the most severe predicted consequence for each variant from Ensembl [111].

Figure S11. Manhattan plot of genome-wide *FineMAV* scores in Admixed Americans and South Asians.
FineMAV scores calculated for genome-wide SNPs from 1000 Genomes Project Phase 3 [37] in: (A) – South Asians (SAS) and (B) – Admixed Americans (AMR). Each dot in the Manhattan plots represents a single SNP plotted according to coordinates in GRCh37.

Figure S12. Signal of selection in the *PRSS53*.
FineMAV scores of variants in the genomic window spanning *PRSS53* are plotted as dots. Genomic positions are given in bp according to GRCh37. The selected

variant (rs201075024) in South Asians (SAS) is marked with a dashed line with the variant (rs11150606) selected in East Asians (EAS) just above it.

Figure S13. Genotypes of putatively introgressed SNPs identified by *FineMAV*.
Rows represent individuals from Phase 3 of the 1000 Genomes Project [37] grouped by population, columns indicate variant sites picked up by *FineMAV* and falling in regions putatively introgressed from archaic hominins [100-103] ordered by genomic location. The associated gene name is given above the SNP ID. The first two rows specify the Neanderthal [130] and Denisova [131] genotypes from high-coverage genomes colored according to genotype: white dot – homozygote for ancestral allele; violet dot – homozygote for derived allele; pink – heterozygote. Human genotypes are denoted by lines (using the same color coding). The bottom panel specifies the *FineMAV* score for each variant.

Figure S14. Fold change between the *FineMAV* scores of gold standard variants and neutral variation across different values of *x* parameter.
The x-axis shows the penalty parameter (x) and the y-axis the fold change between the *FineMAV* values of gold standard variant and the highest scoring nearby neutral variant. Fold-changes were calculated in genomic windows spanning the gold standard genes and are labelled using the gene name. Fold-change below 1 indicates that neutral variant scored higher than the gold standard SNP. Increment of the *x* parameter increases the difference between the *FineMAV* scores of selected and neutral SNPs until it reaches a plateau (observed for *HERC2* and *SLC39A4* in the range examined) and then decreases (observed for *ABCC11* and *ACKR1* in the range examined).

Figure S15. The overall rank of the gold standard variants in the whole-genome *FineMAV* and *DAPxDAF* distributions across different values of *x*.

Improvement of the average rank of the 8 gold standard variants is given against the increasing value of *x* (shown on the x-axis). The improvement was calculated as a fold change between the *FineMAV* or *DAPxDAF* ranks calculated for x_1 and $x_2=x_1+0.5$ (shown on the y-axis). The average rank improves dramatically with increasing *x* until 2.5, and then plateaus (with further decrease above 4 in case of *DAPxDAF* distribution).

Figure S16. Overlap between the top 100 *FineMAV* outliers across different values of *x* parameter.

Numbers show the number of outliers shared between *FineMAV* runs with different values of *x*. A – overlap analysis in Africans; B – overlap analysis in East Asians; C – overlap analysis in Europeans.

Figure S17. Recommended minimal values of *x* for given *n*.

The x-axis shows the number of populations (*n*) and y-axis the penalty parameter (*x*) used in the analyses. This adjustment makes *FineMAV* values comparable across different values of *n*.

Figure S18. Balance between *DAPxDAF* and *CADD* scores across different values of *x* parameter in Africans.

The x-axis shows *DAPxDAF* and y-axis CADD scores across different values of *x* used in the analyses. The dashed line indicates the CADD score threshold of 10.

Green dots indicate the top 100 *FineMAV* outliers. Gold standard variants are labelled in yellow. Increment of *x* removes the green tail of nearly fixed derived alleles of high *CADD* prediction which disappears around *x*=3.

Figure S19. Balance between *DAPxDAF* and *CADD* scores across different values of *x* parameter in East Asians.

The x-axis shows *DAPxDAF* and y-axis CADD scores across different values of *x* used in the analyses. The dashed line indicates the CADD score threshold of 10. Green dots indicate the top 100 *FineMAV* outliers. Gold standard variants are labelled in yellow. Increment of *x* removes the green tail of nearly fixed derived alleles of high *CADD* prediction which disappears around *x*=3.

Figure S20. Balance between *DAPxDAF* and *CADD* scores across different values of *x* parameter in Europeans.

The x-axis shows *DAPxDAF* and y-axis CADD scores across different values of *x* used in the analyses. The dashed line indicates the CADD score threshold of 10. Green dots indicate the top 100 *FineMAV* outliers. Gold standard variants are labelled in yellow. Increment of *x* removes the green tail of nearly fixed derived alleles of high *CADD* prediction which disappears around *x*=3.

Figure S21. Meta-analysis of published genome-wide positive selection scans.

(A) – Plot of Selection Support Index (*SSI*) scores for the positively selected genes in the published literature against the number of genes with this score; *SSI* score is also illustrated by the circle colour, and gene count by the circle size. (B) – Manhattan plot of the top ~6% putatively selected loci meeting the threshold of

SSI score ≥ 0.09; each dot represents a gene midpoint; the cluster of genes on chromosome 2 underlying lactose tolerance is boxed. (C) – An expanded view of the lactase persistence signal showing strong signature of positive selection that extends over a large genomic region; each dot represents the midpoint of a gene surrounding *LCT*; genes are shown as rectangular boxes in the gene track plotted below the x-axis displaying their chromosomal positions in GRCh37.

Figure S22. Distribution of *FineMAV* scores in *SSI* outlier genes.
Comparison of the distribution of *FineMAV* scores of top SNPs falling in *SSI* outlier genes and their 50 kb surrounding regions (*SSI* ≥ 0.18 which corresponds to ~1% of top genes) with analogical distribution for random genes. The two distributions were found to be significantly different (p-value = 1.89×10^{-5}). Dashed vertical lines indicate *FineMAV* cutoffs to include top 100 variants in each continental population.

Figure S1

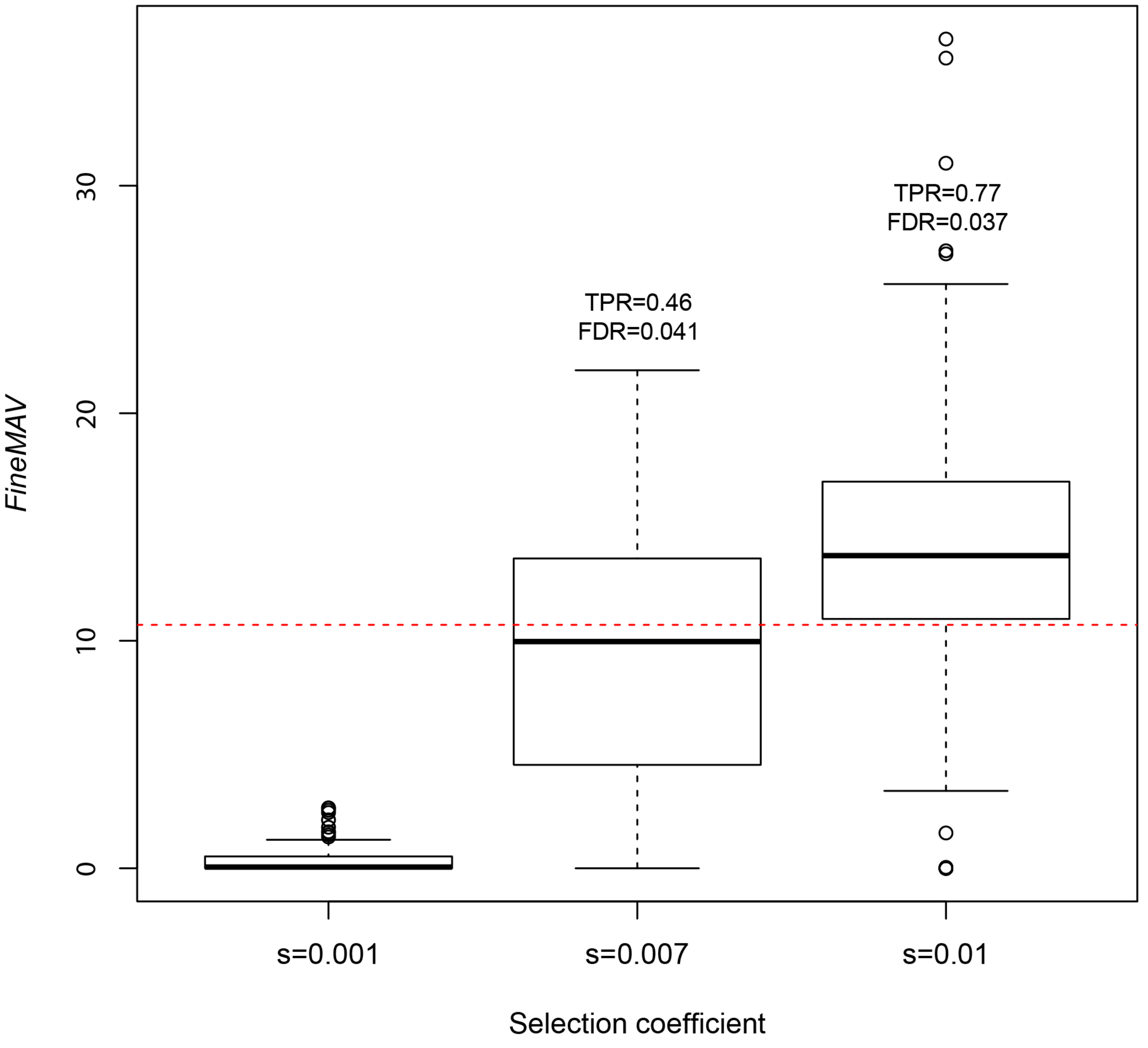

TPR=0.46
FDR=0.041
TPR=0.77
FDR=0.037
0
10
20
30
FineMAV
s=0.001
s=0.007
s=0.01
Selection coefficient

Figure S2

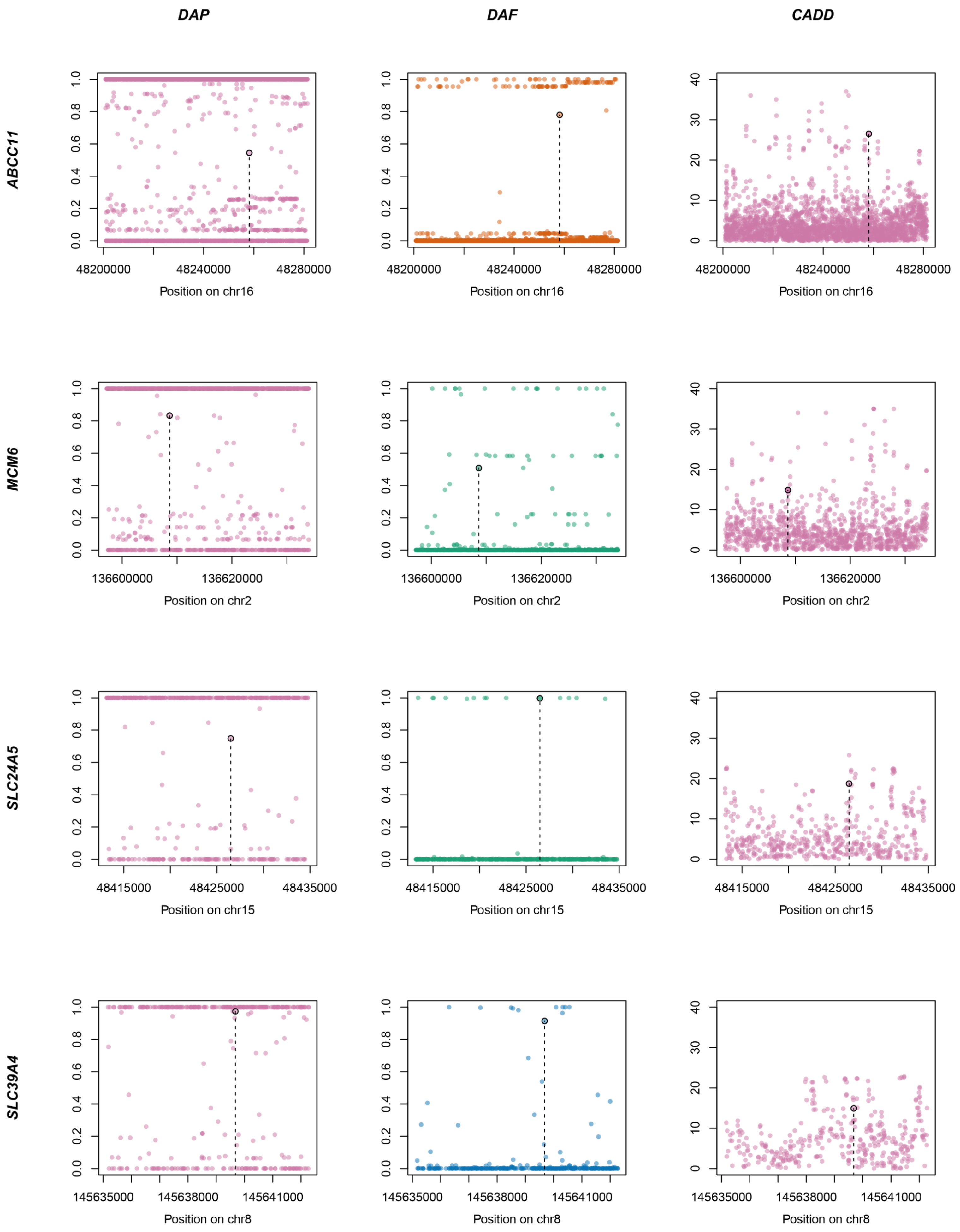

DAP
DAF
CADD
ABCC11
MCM6
SLC24A5
SLC39A4
Position on chr16
Position on chr2
Position on chr15
Position on chr8
48200000
48240000
48280000
136600000
136620000
48415000
48425000
48435000
145635000
145638000
145641000

Figure S3

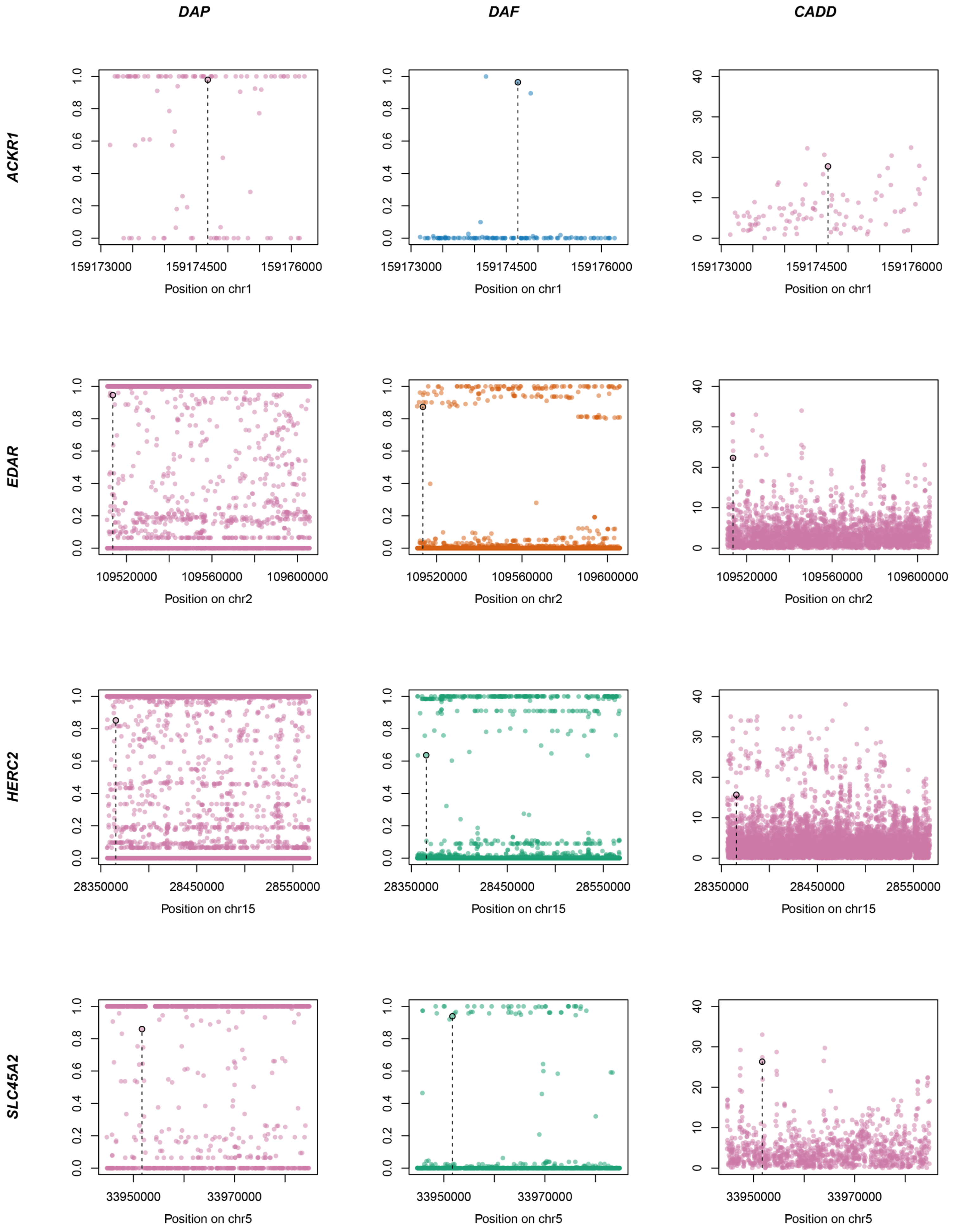
DAP
DAF
CADD
ACKR1
EDAR
HERC2
SLC45A2
159173000
159174500
159176000
Position on chr1
109520000
109560000
109600000
Position on chr2
28350000
28450000
28550000
Position on chr15
33950000
33970000
Position on chr5

Figure S4

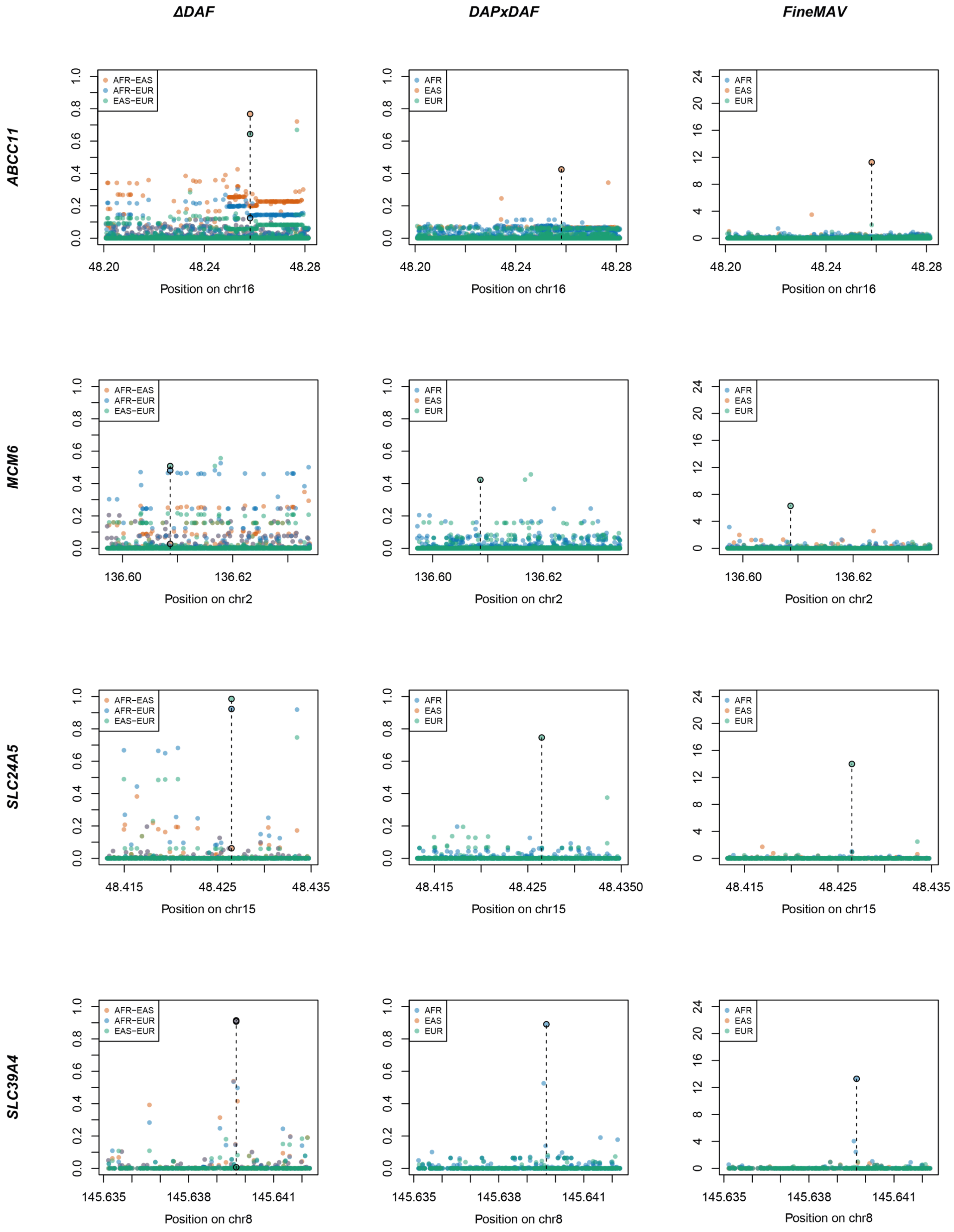

ΔDAF
DAPxDAF
FineMAV
ABCC11
MCM6
SLC24A5
SLC39A4
AFR−EAS
AFR−EUR
EAS−EUR
AFR
EAS
EUR
Position on chr16
Position on chr2
Position on chr15
Position on chr8

Figure S5

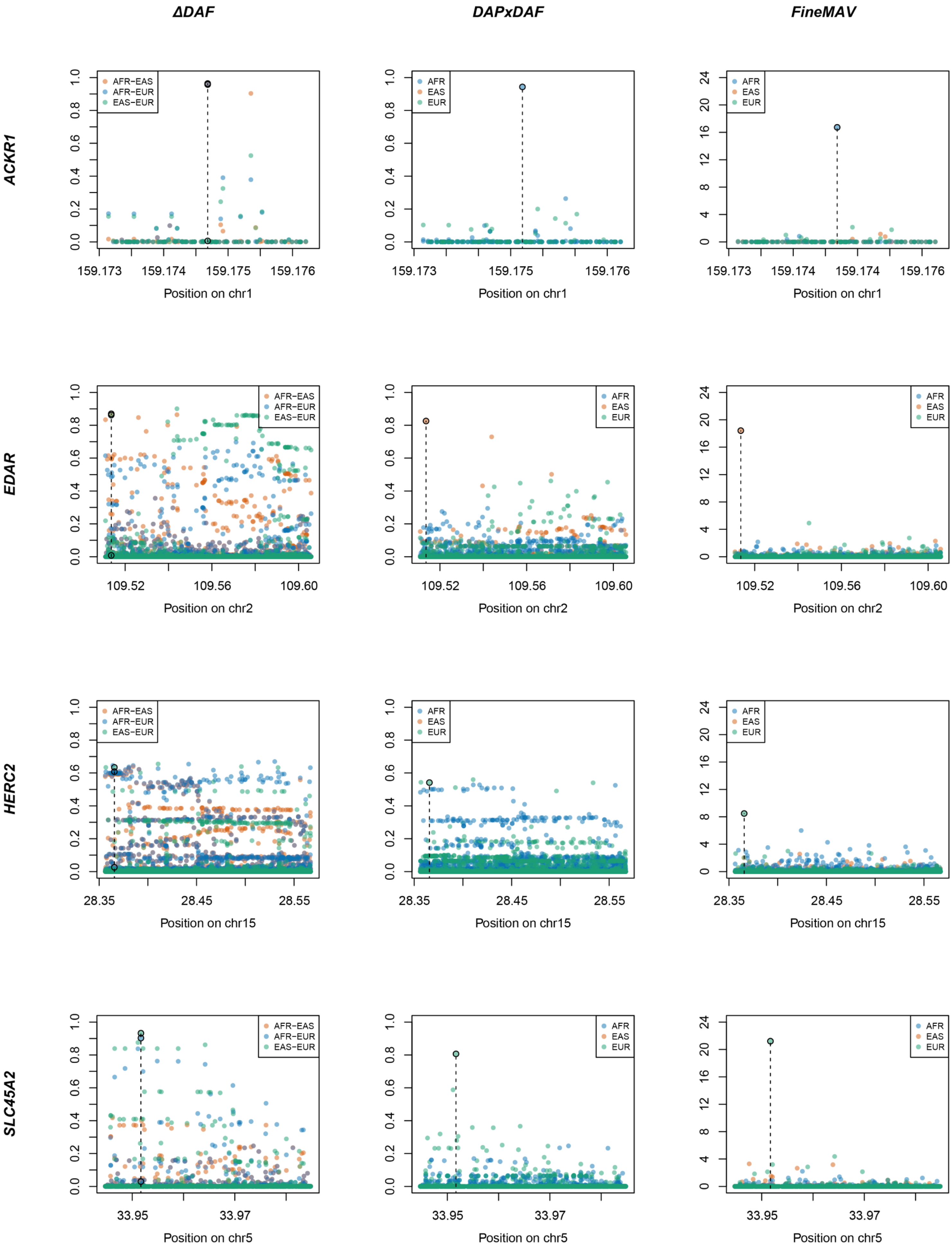
ΔDAF
DAPxDAF
FineMAV
ACKR1
EDAR
HERC2
SLC45A2
AFR−EAS
AFR−EUR
EAS−EUR
AFR
EAS
EUR
Position on chr1
Position on chr2
Position on chr15
Position on chr5
159.173
159.174
159.175
159.176
109.52
109.56
109.60
28.35
28.45
28.55
33.95
33.97
0.0
0.2
0.4
0.6
0.8
1.0
0
4
8
12
16
20
24

Figure S6

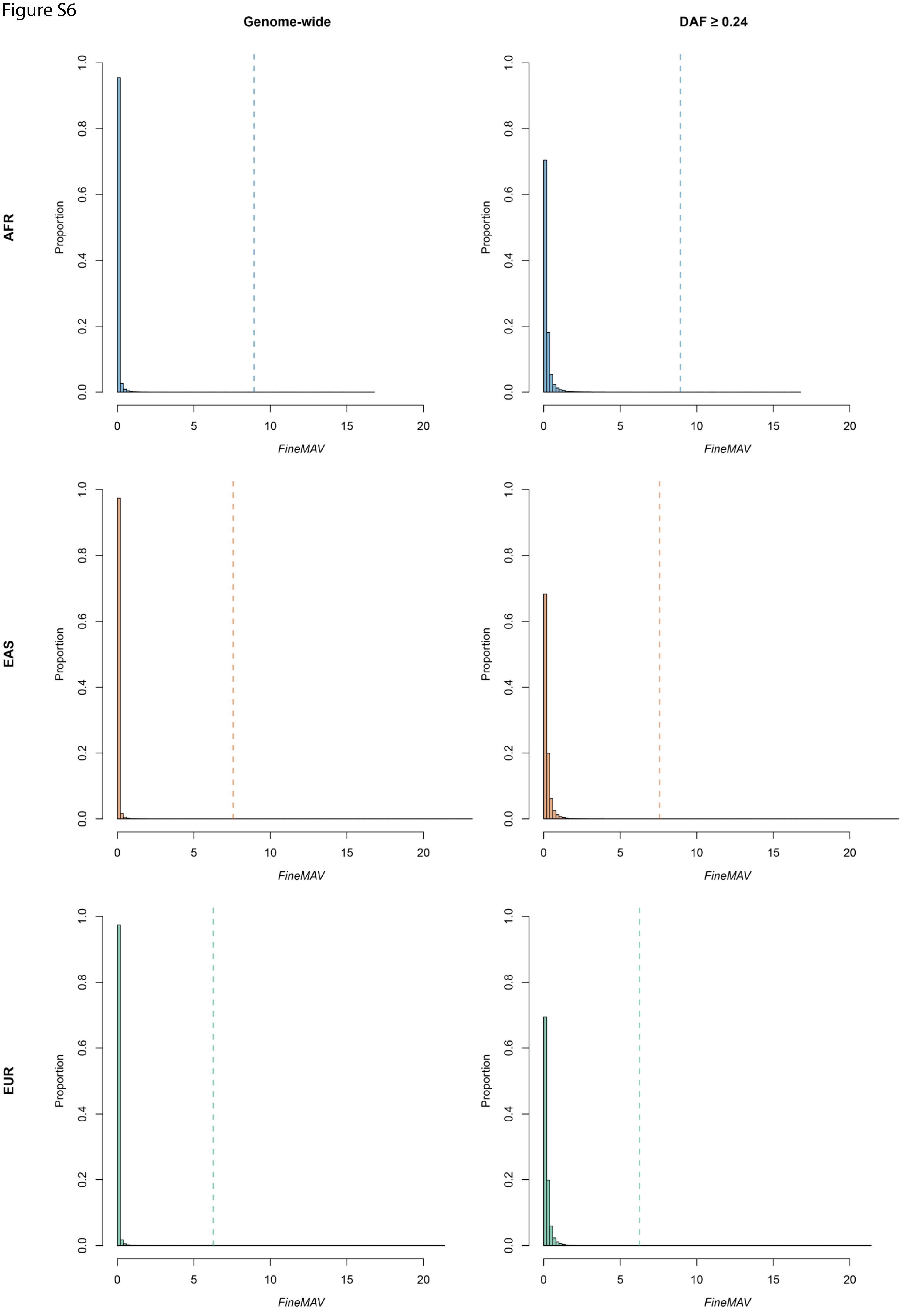

Genome-wide
DAF ≥ 0.24
AFR
EAS
EUR
Proportion
FineMAV
0.0
0.2
0.4
0.6
0.8
1.0
0
5
10
15
20

Figure S7

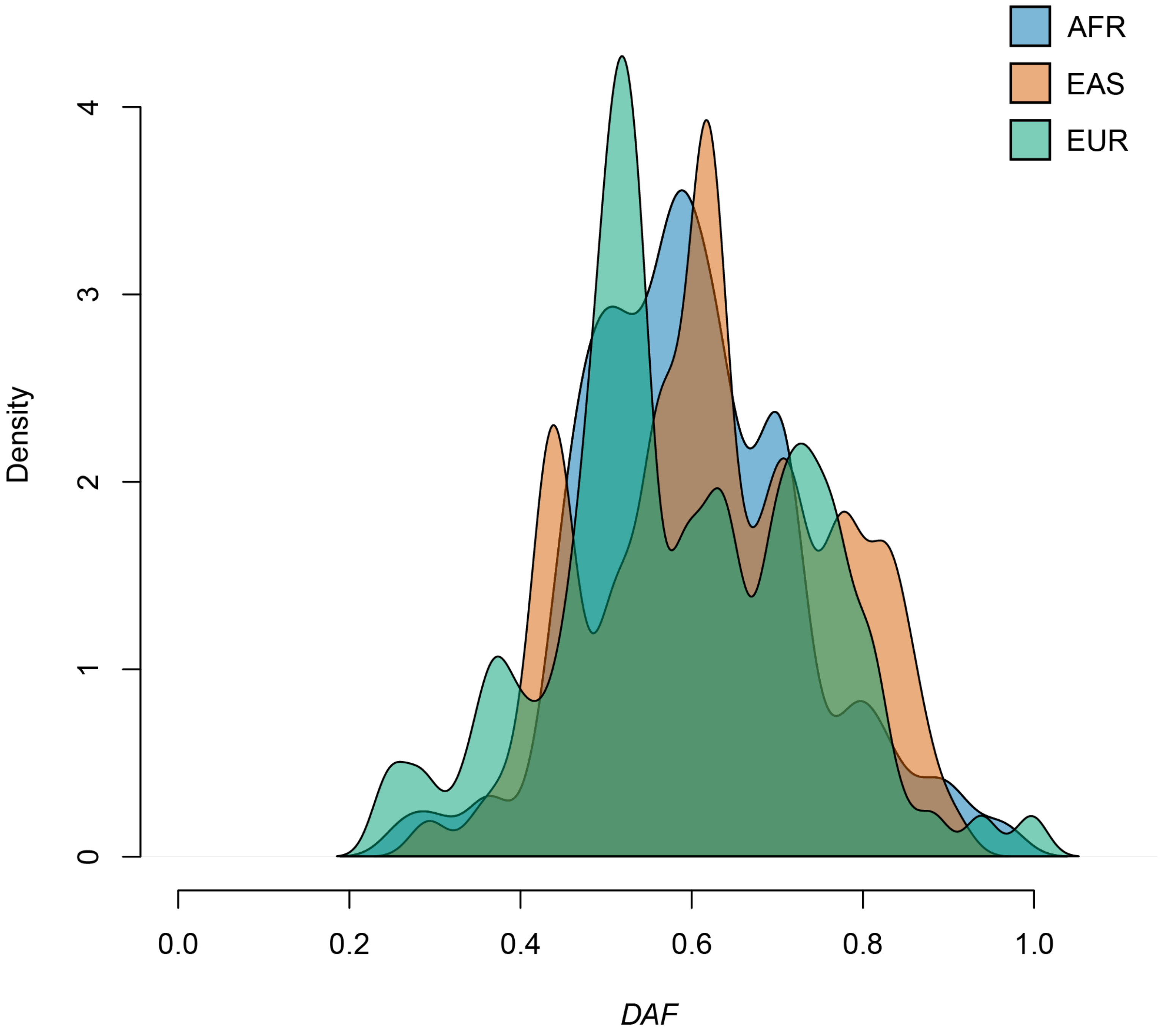

Figure S8

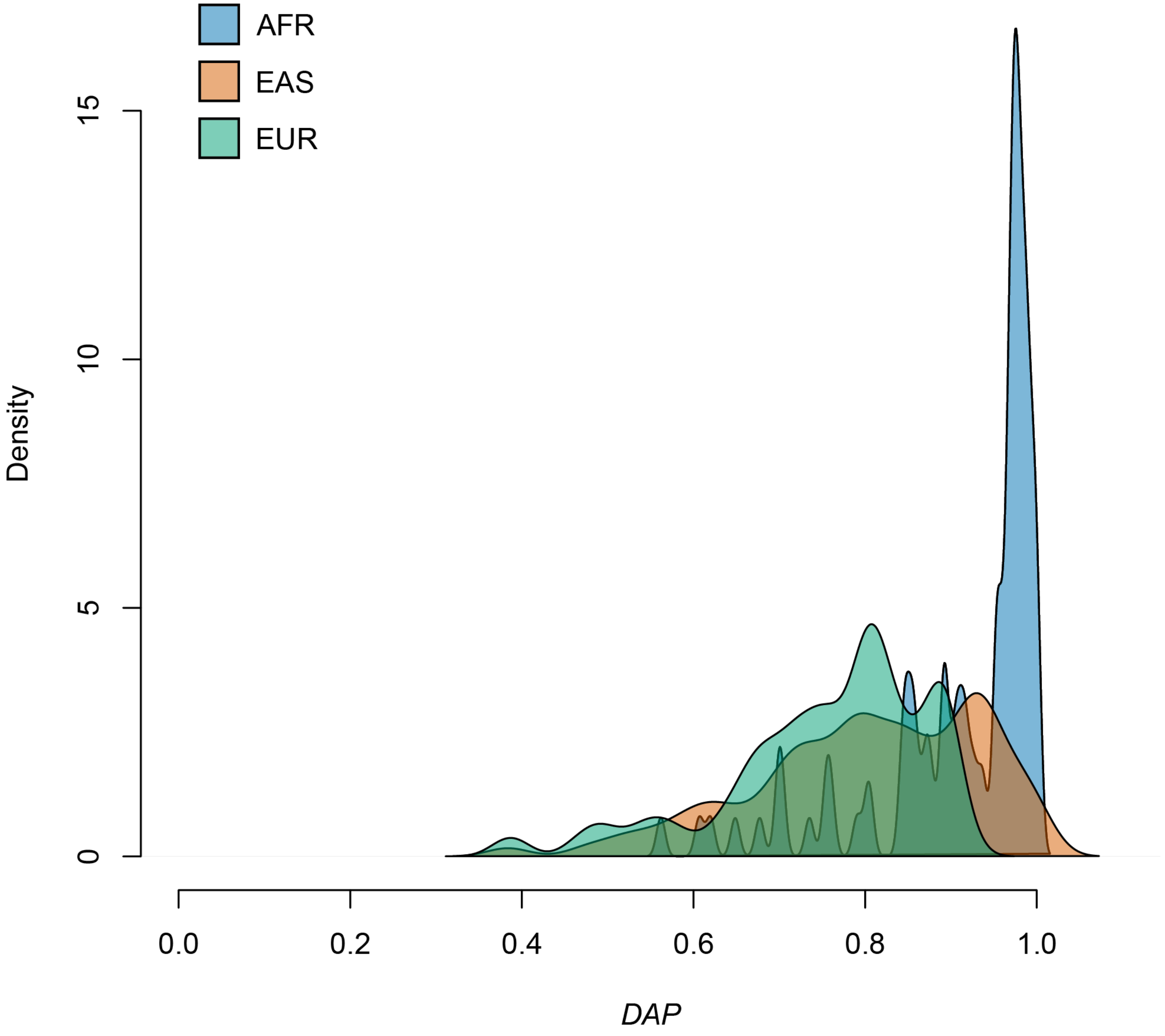

Figure S9

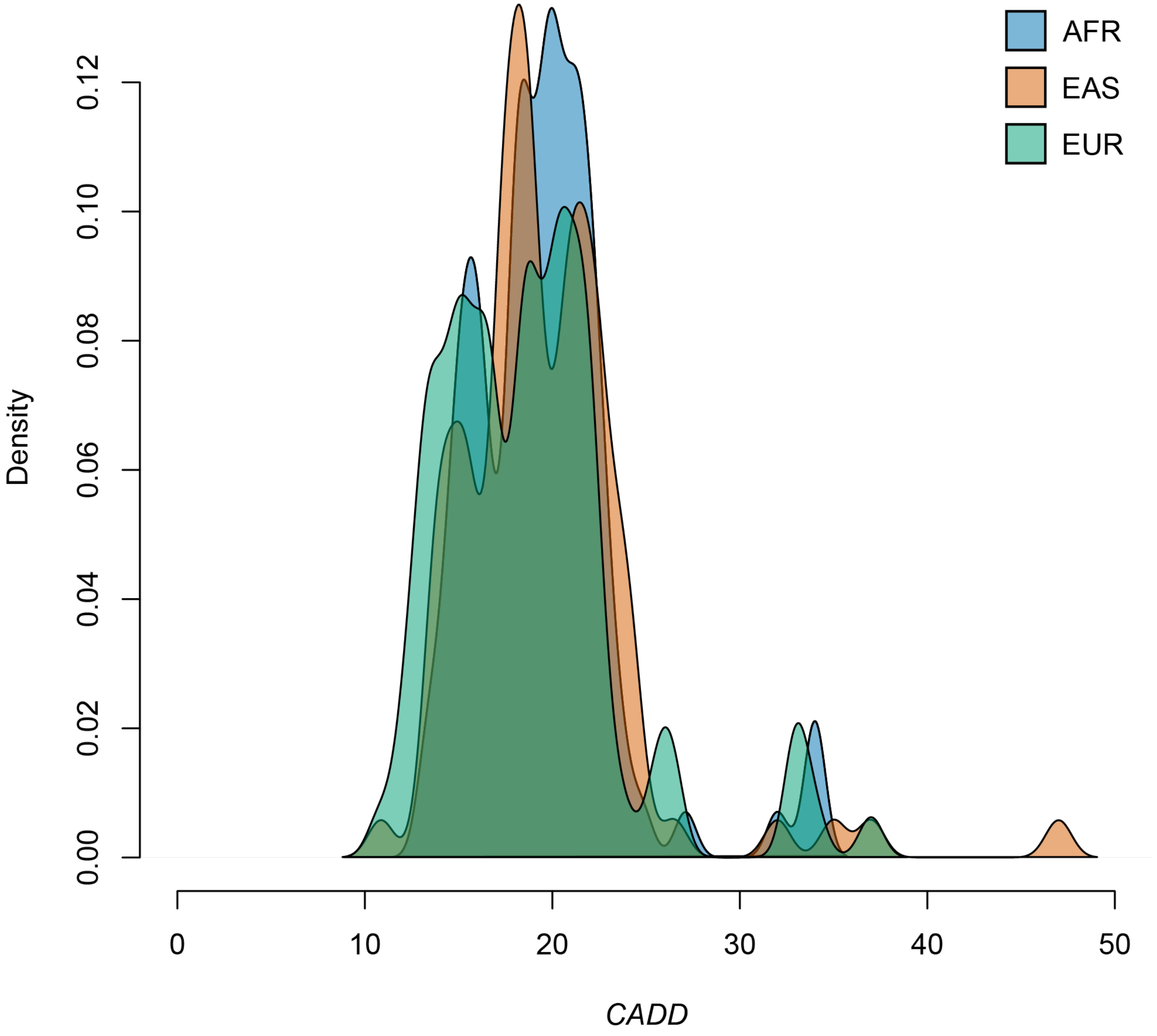
AFR
EAS
EUR
Density
0.00
0.02
0.04
0.06
0.08
0.10
0.12
0
10
20
30
40
50
CADD

Figure S10

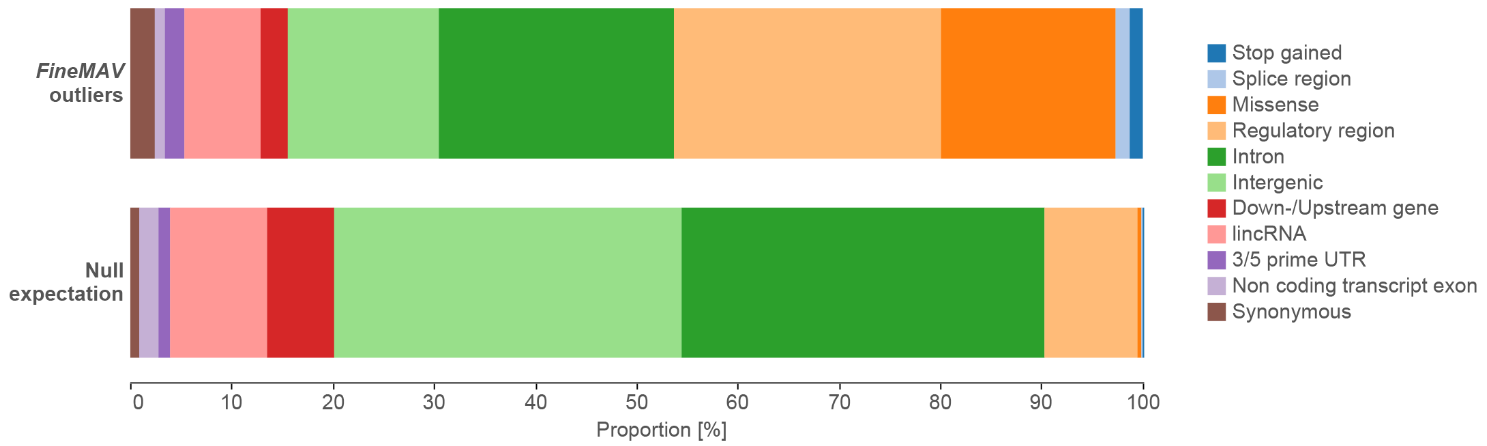

FineMAV outliers
Null expectation
0
10
20
30
40
50
60
70
80
90
100
Proportion [%]
Stop gained
Splice region
Missense
Regulatory region
Intron
Intergenic
Down-/Upstream gene
lincRNA
3/5 prime UTR
Non coding transcript exon
Synonymous

Figure S11

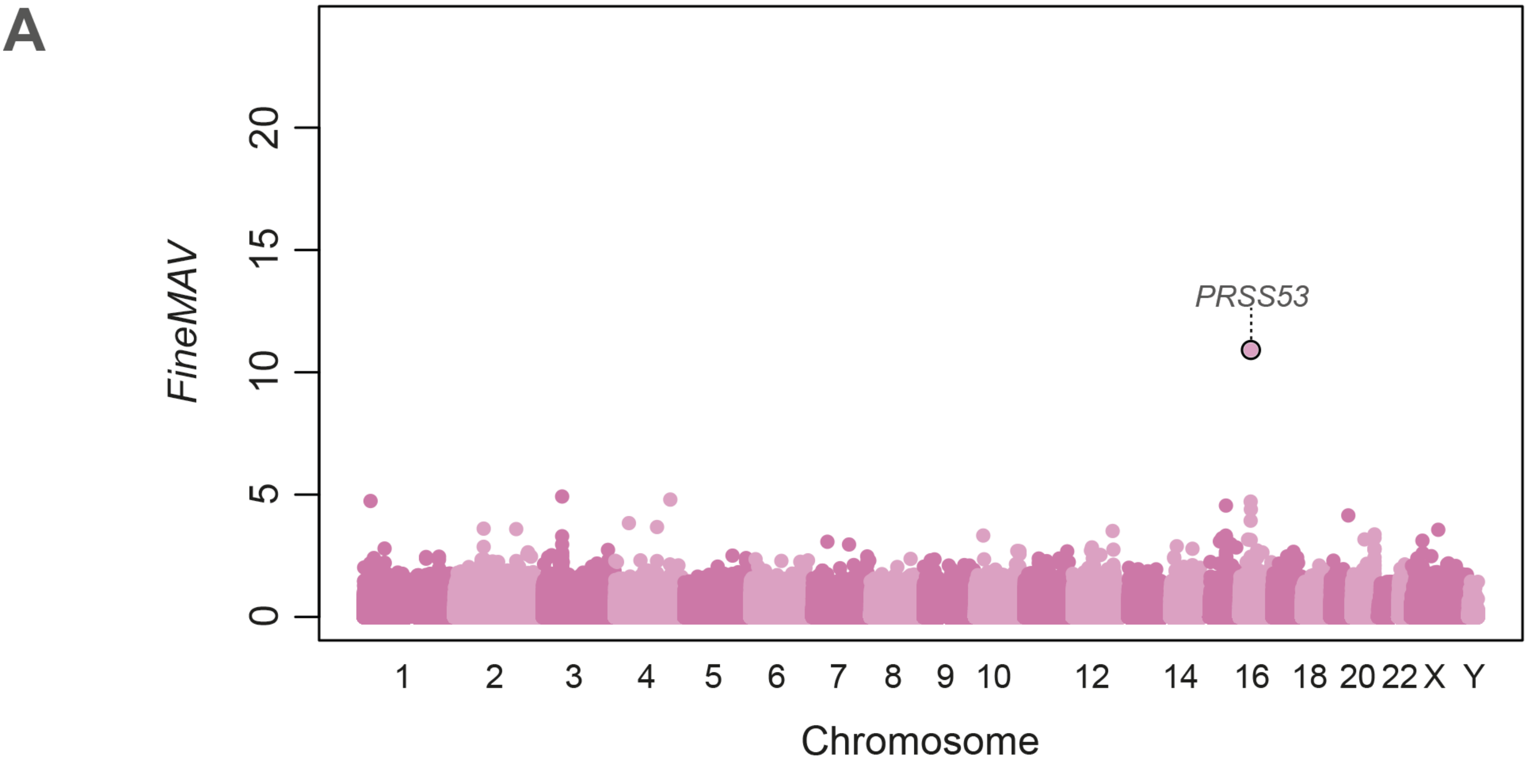
A
FineMAV
0
5
10
15
20
PRSS53
1
2
3
4
5
6
7
8
9
10
12
14
16
18
20
22
X
Y
Chromosome

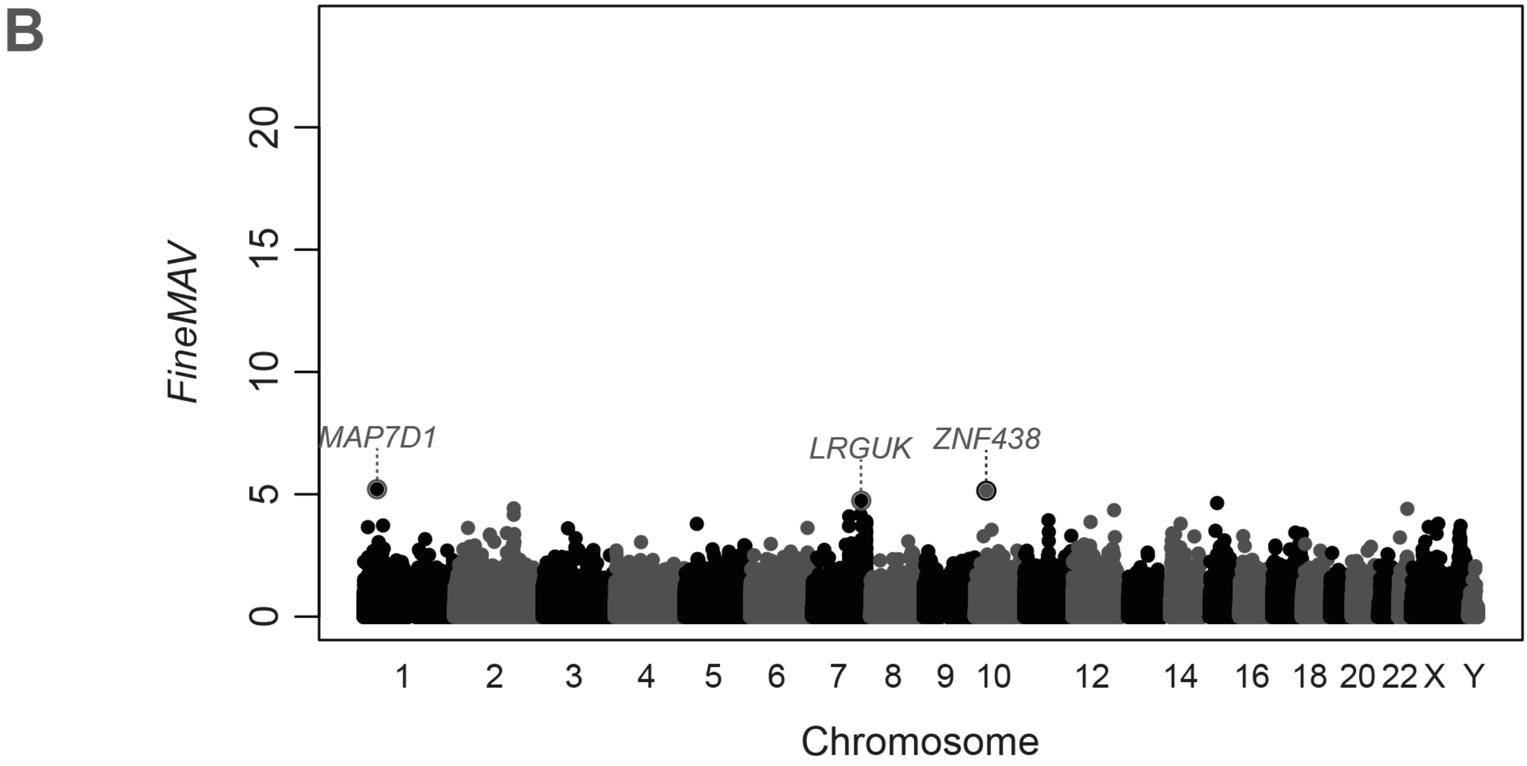
B
FineMAV
0
5
10
15
20
MAP7D1
LRGUK
ZNF438
1
2
3
4
5
6
7
8
9
10
12
14
16
18
20
22
X
Y
Chromosome

Figure S12

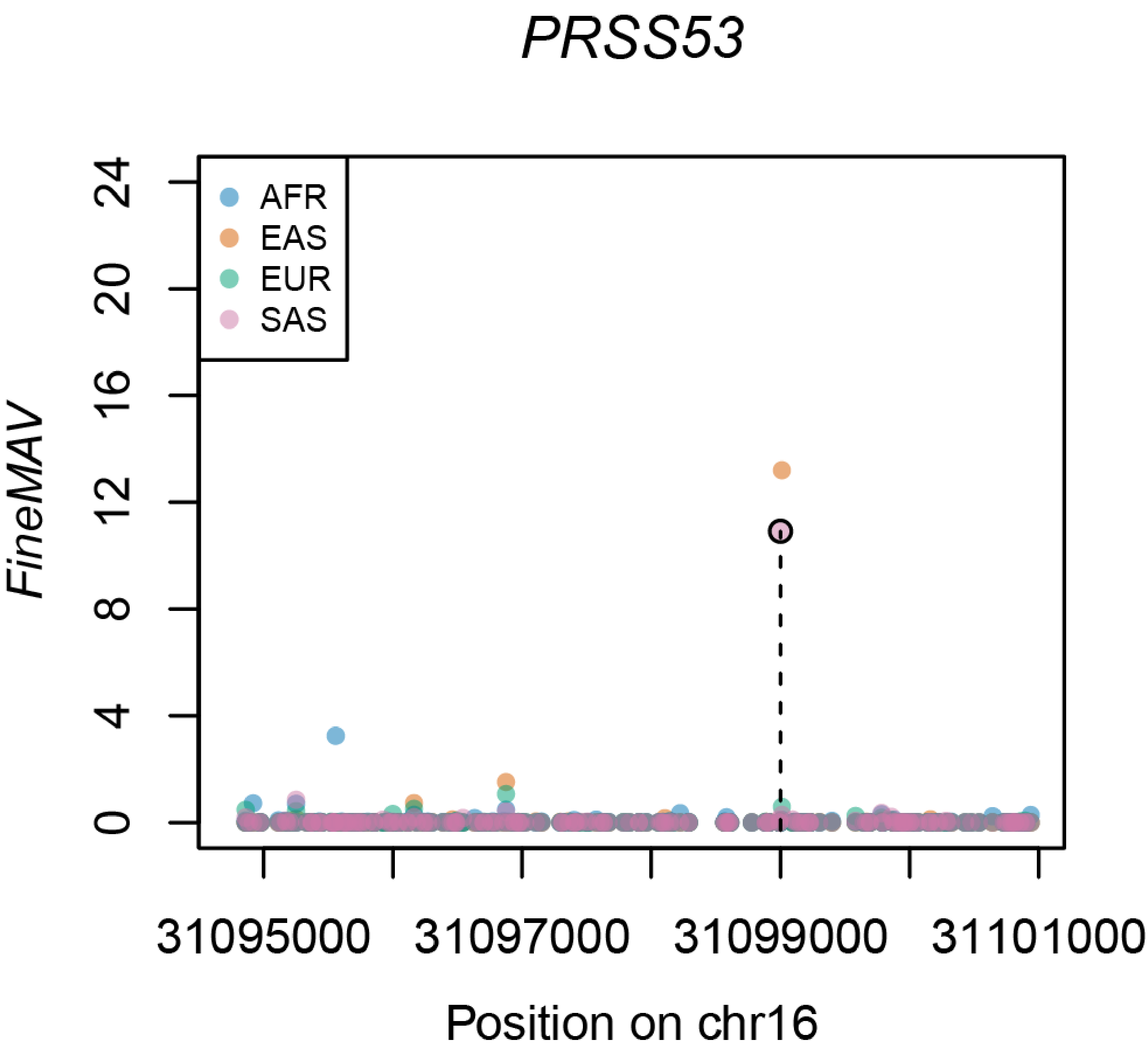

PRSS53
AFR
EAS
EUR
SAS
FineMAV
0
4
8
12
16
20
24
31095000
31097000
31099000
31101000
Position on chr16

Figure S13

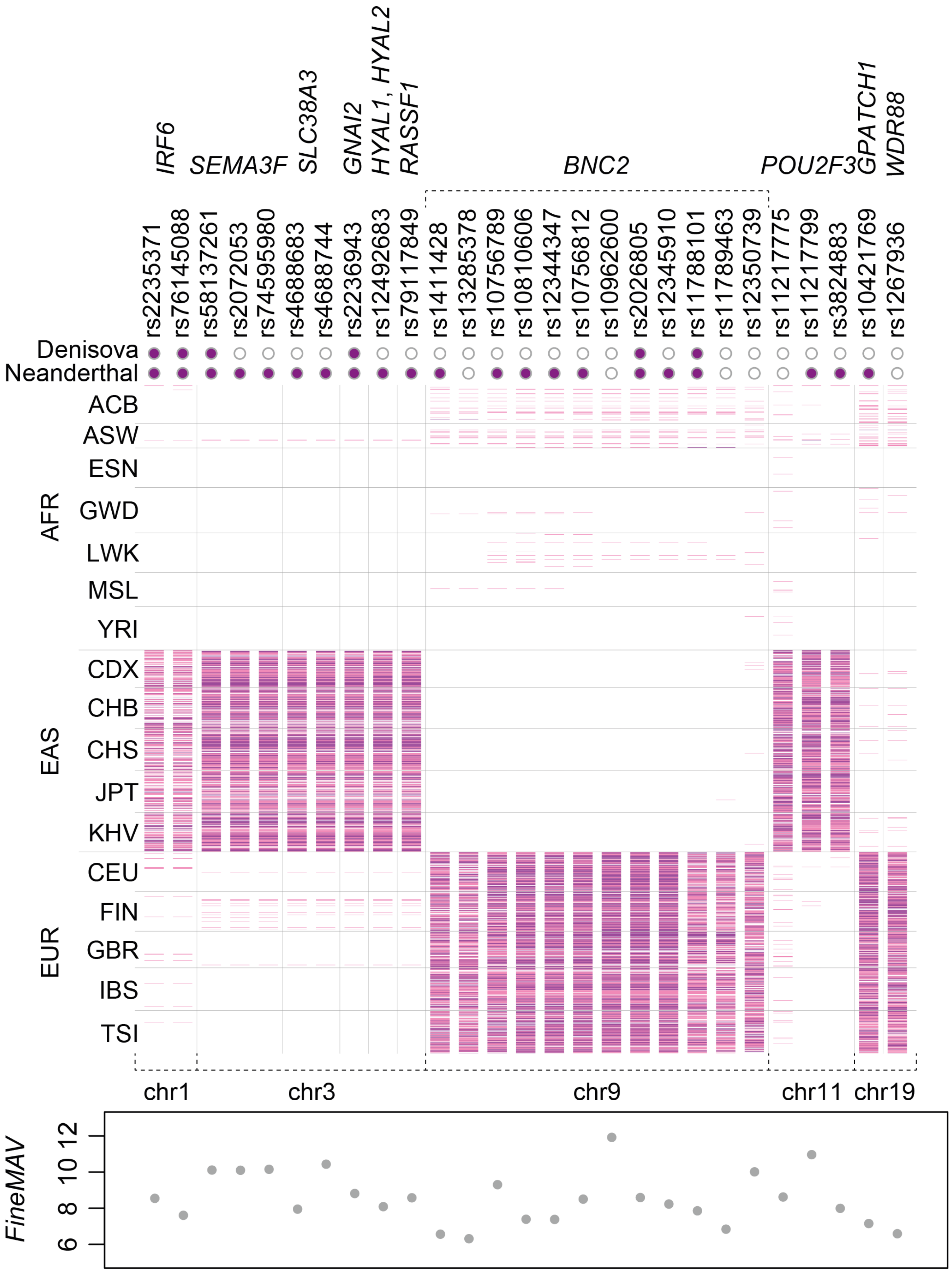

IRF6
SEMA3F
SLC38A3
GNAI2
HYAL1, HYAL2
RASSF1
BNC2
POU2F3
GPATCH1
WDR88
rs2235371
rs76145088
rs58137261
rs2072053
rs74595980
rs4688683
rs4688744
rs2236943
rs12492683
rs79117849
rs1411428
rs13285378
rs10756789
rs10810606
rs12344347
rs10756812
rs10962600
rs2026805
rs12345910
rs11788101
rs11789463
rs12350739
rs11217775
rs11217799
rs3824883
rs10421769
rs1267936
Denisova
Neanderthal
ACB
ASW
ESN
GWD
LWK
MSL
YRI
AFR
CDX
CHB
CHS
JPT
KHV
EAS
CEU
FIN
GBR
IBS
TSI
EUR
chr1
chr3
chr9
chr11
chr19
FineMAV
6
8
10
12

Figure S14

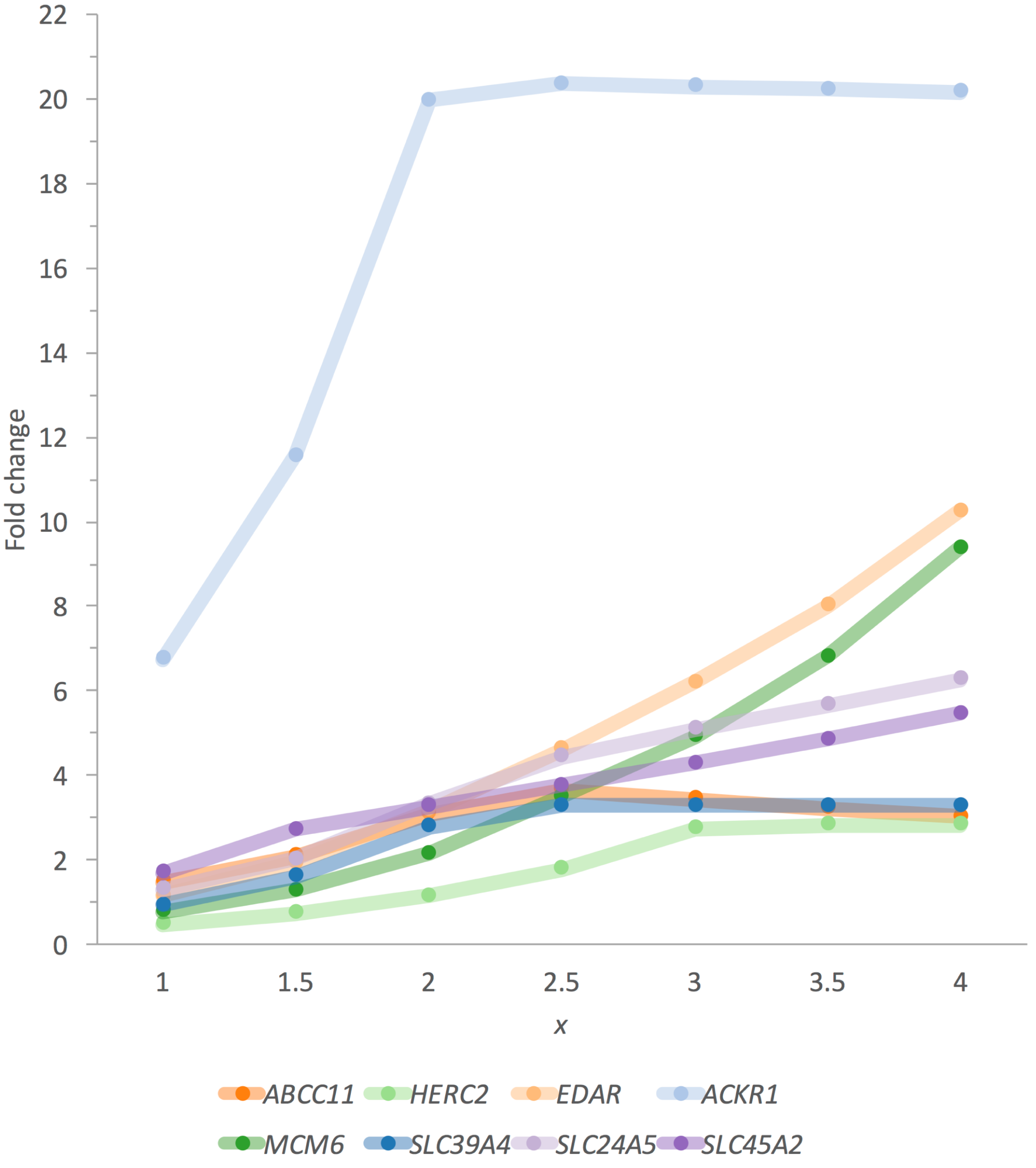

22
20
18
16
14
12
10
8
6
4
2
0
Fold change
1
1.5
2
2.5
3
3.5
4
x
ABCC11
HERC2
EDAR
ACKR1
MCM6
SLC39A4
SLC24A5
SLC45A2

Figure S15

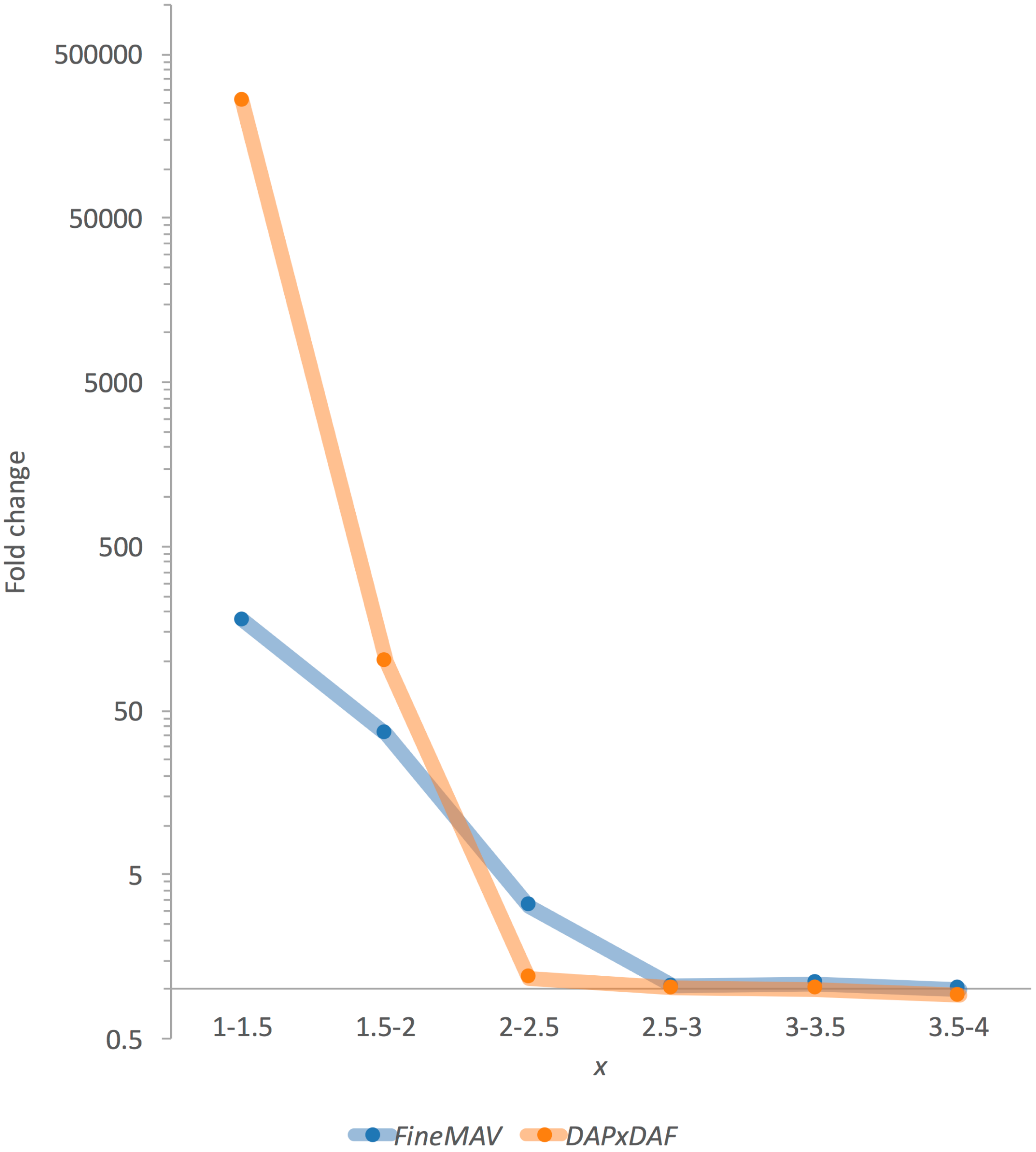

500000
50000
5000
500
50
5
0.5
Fold change
1-1.5
1.5-2
2-2.5
2.5-3
3-3.5
3.5-4
x
FineMAV
DAPxDAF

Figure S16

A

x	1	1.5	2	2.5	3	3.5	4
1		86	17	0	0	0	0
1.5	86		30	12	12	12	12
2	17	30		81	79	75	72
2.5	0	12	81		97	92	89
3	0	12	79	97		95	92
3.5	0	12	75	92	95		97
4	0	12	72	89	92	97	

B

x	1	1.5	2	2.5	3	3.5	4
1		76	23	3	2	2	2
1.5	76		42	14	12	12	11
2	23	42		62	57	52	49
2.5	3	14	62		90	84	79
3	2	12	57	90		94	88
3.5	2	12	52	84	94		94
4	2	11	49	79	88	94	

C

x	1	1.5	2	2.5	3	3.5	4
1		84	31	5	1	1	1
1.5	84		44	12	6	6	6
2	31	44		62	47	46	43
2.5	5	2	62		84	80	76
3	1	6	47	84		95	91
3.5	1	6	46	80	95		96
4	1	6	43	76	91	96	

Figure S17

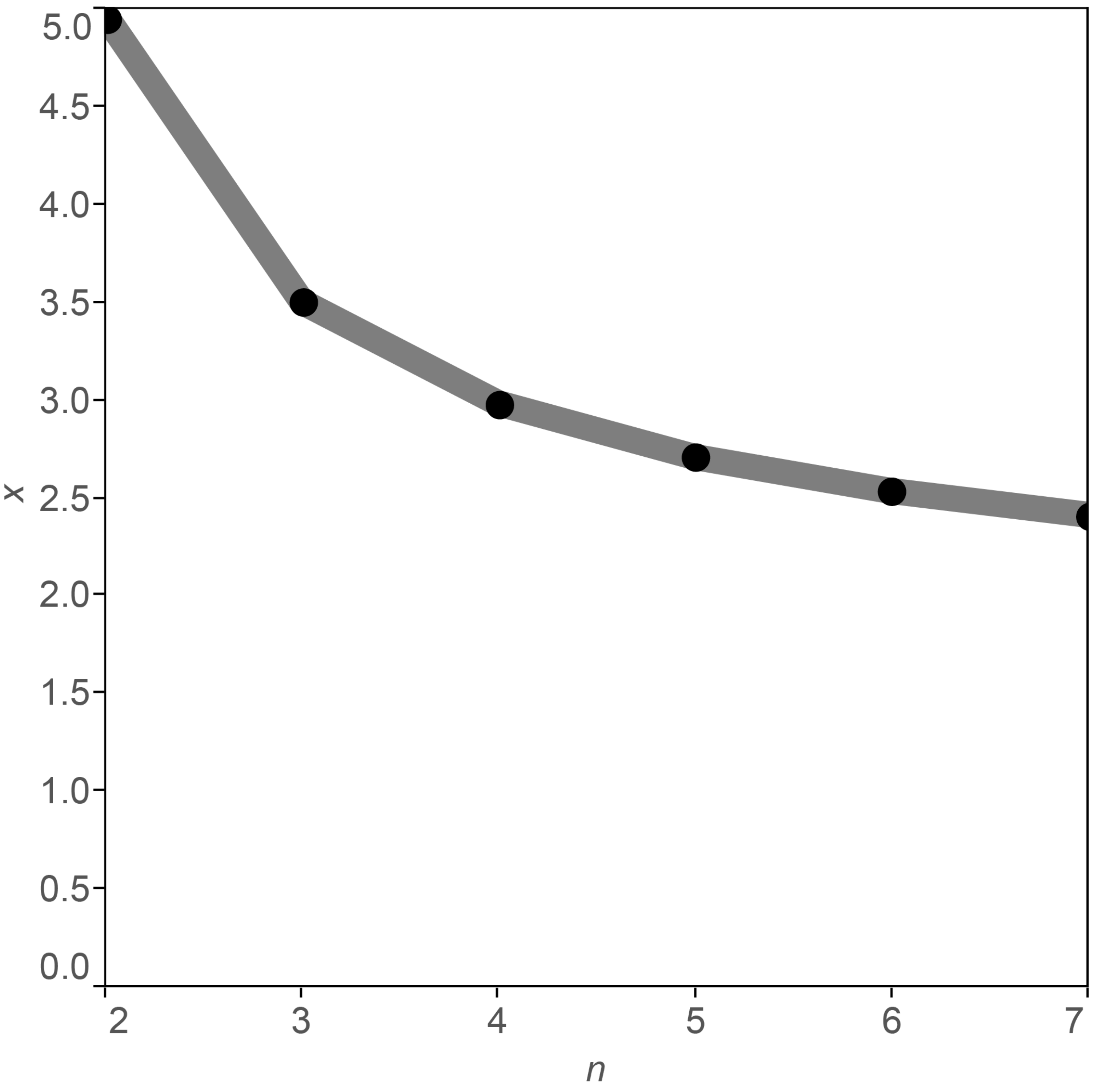

Figure S18

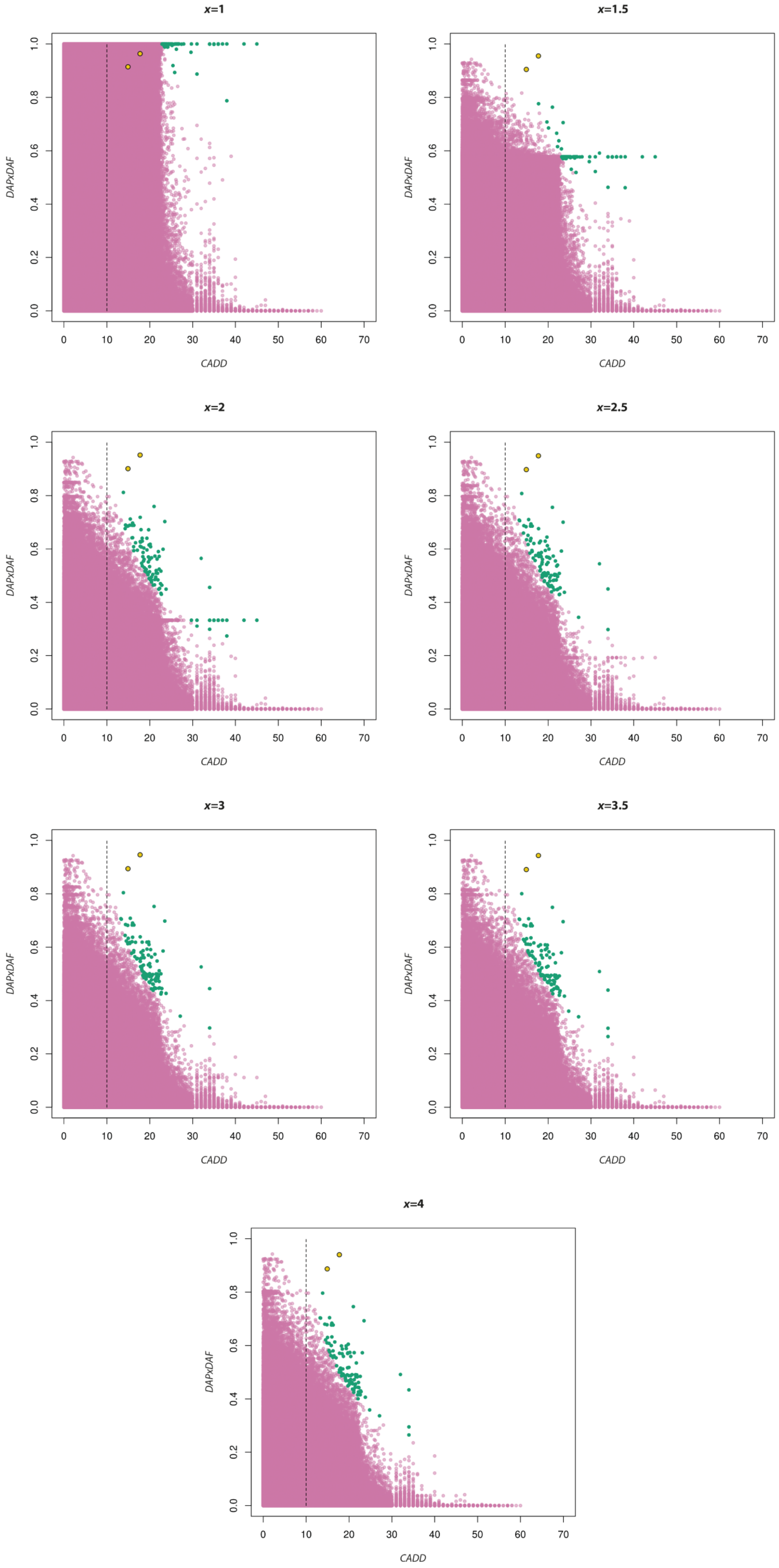
x=1
x=1.5
x=2
x=2.5
x=3
x=3.5
x=4
DAPxDAF
CADD
0.0
0.2
0.4
0.6
0.8
1.0
0
10
20
30
40
50
60
70

Figure S19

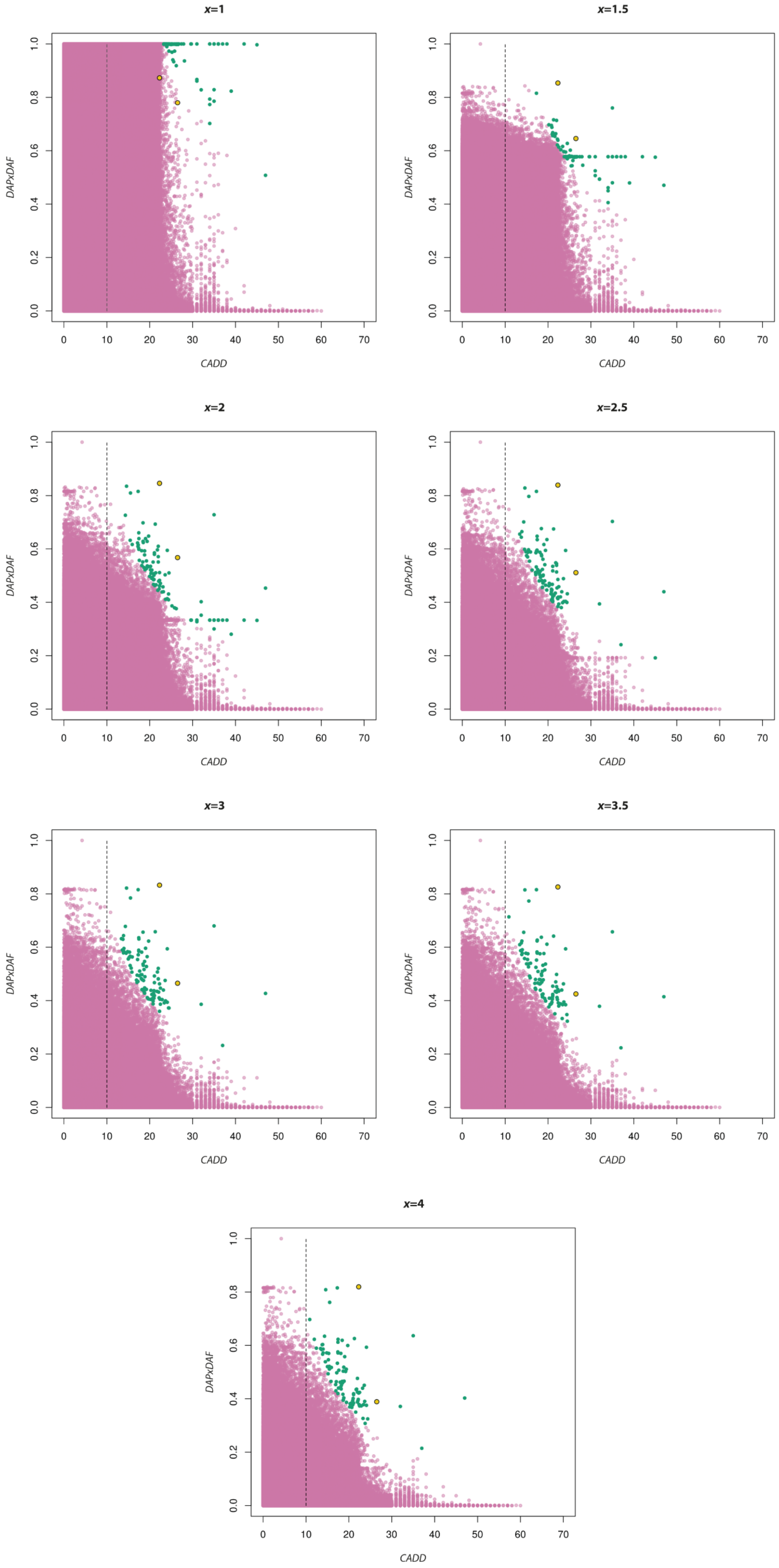
x=1
x=1.5
x=2
x=2.5
x=3
x=3.5
x=4
DAPxDAF
CADD
0.0
0.2
0.4
0.6
0.8
1.0
0
10
20
30
40
50
60
70

Figure S20

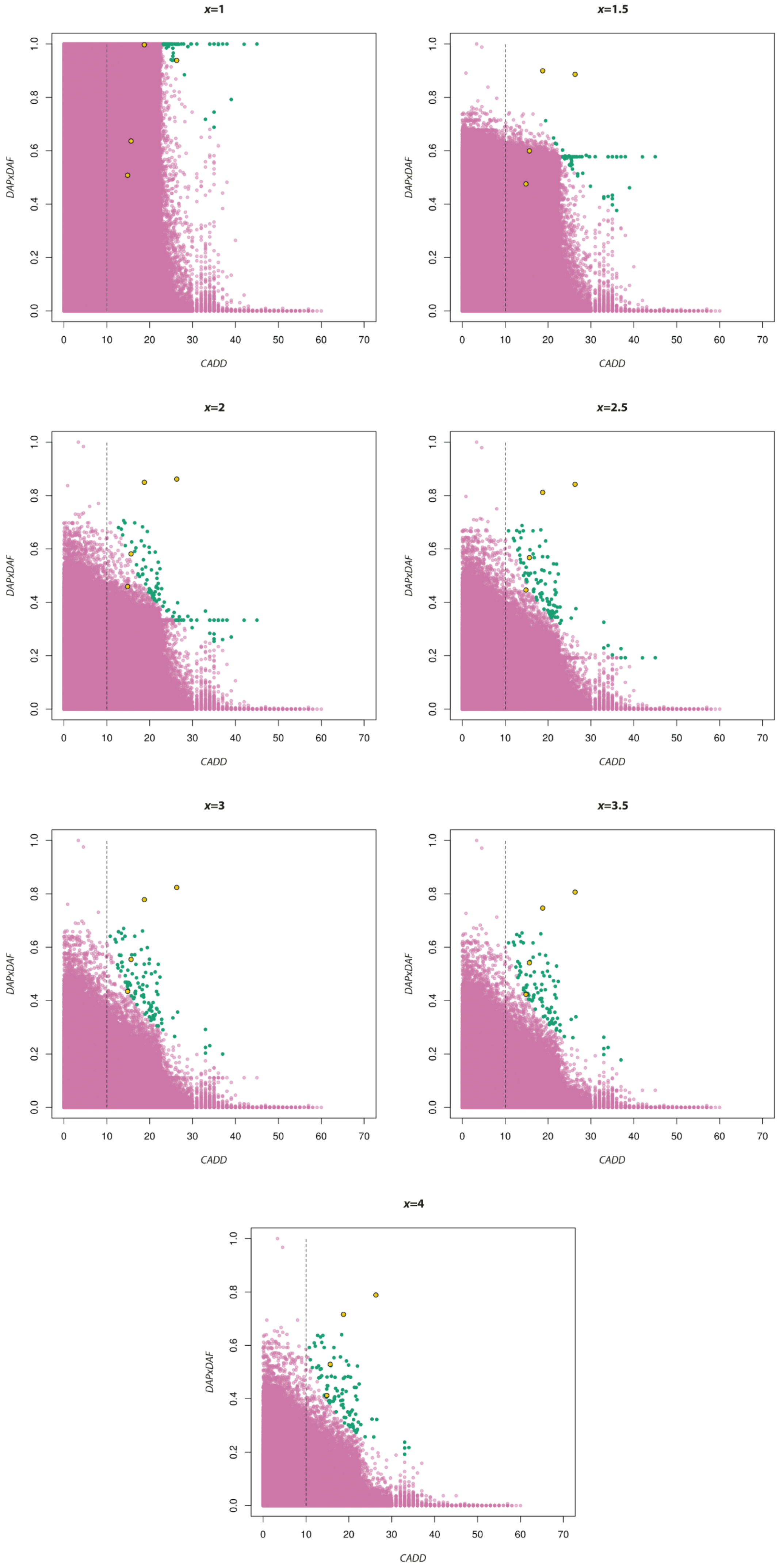

x=1
x=1.5
x=2
x=2.5
x=3
x=3.5
x=4
DAPxDAF
CADD
0.0 0.2 0.4 0.6 0.8 1.0
0 10 20 30 40 50 60 70

Figure S21

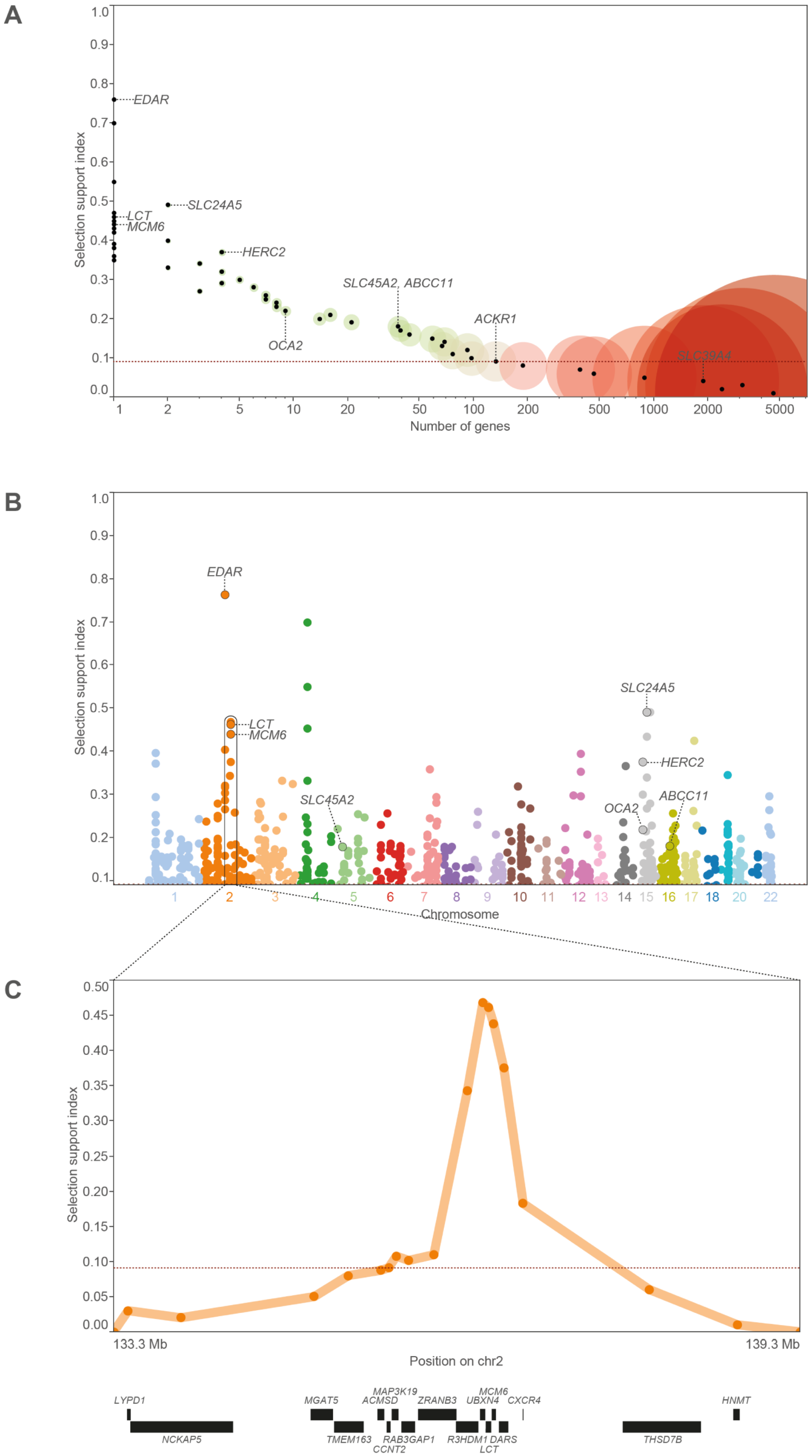
A
EDAR
SLC24A5
LCT
MCM6
HERC2
SLC45A2, ABCC11
OCA2
ACKR1
SLC39A4
Selection support index
Number of genes
B
EDAR
LCT
MCM6
SLC45A2
SLC24A5
HERC2
ABCC11
OCA2
Chromosome
C
Selection support index
133.3 Mb
139.3 Mb
Position on chr2
LYPD1
NCKAP5
MGAT5
TMEM163
MAP3K19
ACMSD
CCNT2
RAB3GAP1
ZRANB3
R3HDM1
MCM6
UBXN4
LCT
DARS
CXCR4
THSD7B
HNMT

Figure S22

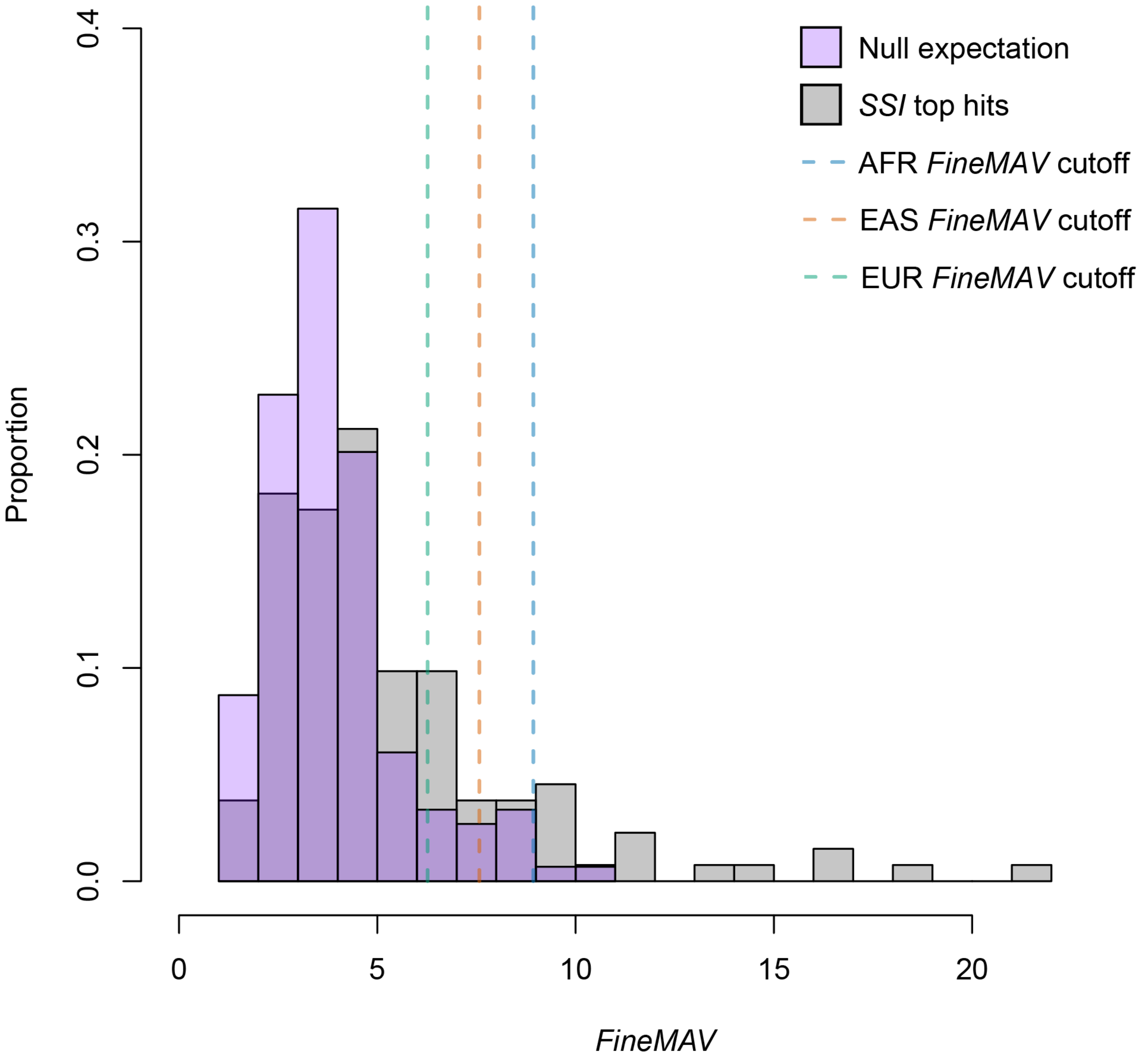

Null expectation
SSI top hits
AFR FineMAV cutoff
EAS FineMAV cutoff
EUR FineMAV cutoff
Proportion
0.0
0.1
0.2
0.3
0.4
0
5
10
15
20
FineMAV
