# Supplementary material for: FineMAV: prioritizing candidate genetic variants driving local adaptations in human populations
Source: Genome Biol. 2018 Jan 17;19:5. doi: 10.1186/s13059-017-1380-2 (PMC5771147; doi:10.1186/s13059-017-1380-2)
Supplement: Supplementary file 2 — Description of meta-analysis, enrichment analyses, and novel candidates found in this study. (DOCX 182 kb) [file 13059_2017_1380_MOESM2_ESM.docx]

***FineMAV*: Prioritizing candidate genetic variants driving local adaptations in human populations**

Michal Szpak,^1^* Massimo Mezzavilla,^1,2^ Qasim Ayub,^1,3^ Yuan Chen,^1^ Yali Xue,^1^ Chris Tyler-Smith^1^**

^1^ Wellcome Trust Sanger Institute, Wellcome Genome Campus, Hinxton CB10 1SA, UK.

^2^ Division of Experimental Genetics, Sidra Medical and Research Center, Doha, Qatar.

^3^ Present Address: School of Science, Monash University Malaysia, Bandar Sunway, Selangor Darul Ehsan, Malaysia.

* ms30@sanger.ac.uk

** cts@sanger.ac.uk

**Additional file 2**

**Meta-analysis of previous selection scans**

Since there is a large literature on positive selection in humans, we performed a meta-analysis of previous studies at the gene level to obtain a summary of the field, which was used to compare with the *FineMAV* results. Many diverse approaches have been used to search for positive selection footprints, most based on a single characteristic left by a hard sweep, although emerging composite likelihood methods combine multiple lines of evidence (as a strong hard sweep should leave multiple signatures) [[1](#_ENREF_1)]. Each method picks up a slightly different signal and has its own strengths and weaknesses [[2](#_ENREF_2)], so combining complementary methods should increase the chance of finding truly selected loci, as selected loci reported by multiple studies are more likely to be real [[1](#_ENREF_1)].

We examined the concordance of all available genome-wide screens for positive selection (published until September 2014), focusing on recent or ongoing positive selection, i.e. adaptations following the ‘Out-of-Africa’ dispersal that have not swept to fixation in the species yet (incomplete sweeps or so-called microevolution). It is important to carefully curate ‘input data’ by selecting studies investigating the same mode of selection (identifying selective events of the same age and stage of selective sweep) from comparable genome-wide datasets in such an analysis [[1](#_ENREF_1)]. Therefore, we searched the PubMed publication database (‘positive selection’ enquiry) for studies using (i) tests based on intra-species polymorphism (excluding cross-species comparisons) and (ii) genome-wide sequencing or genotyping data (iii) across at least three main continental groups (Africans, East Asians and Europeans). This search yielded 26 genome-wide selection scans [[3-28](#_ENREF_3)] complemented with an unpublished SFS analysis of 1000 Genomes Project Phase 1 [[29](#_ENREF_29)]. These were grouped into four methodological categories: (i) population differentiation (*Diff*), (ii) long haplotypes (*LD*), (iii) site frequency spectra (*SFS*) and (iv) composite likelihood methods (*Comp*). All reported findings were translated into gene-level nomenclature using Ensembl annotation [[30](#_ENREF_30)]. Genes reported only by a single study were excluded at this stage.

Since one particular method of looking for evidence of selection might be more abundant in the published literature than others, its results might outweigh other methods in a simple summation of the evidence and inappropriately dominate a meta-analysis. To avoid this bias and obtain a balanced view based on all four methods, we developed a correction to control for the proportion of studies that are not independent. We first calculated a per-gene selection confidence level within each methodological category (ranging from 0 for genes not reported by any study within that category, to 1 for genes supported by all selection scans employing that detection method). We then calculated a Selection Support Index (*SSI*) by first obtaining the mean of the squares of the selection confidence levels on a per-gene basis. This would penalize genes moderately supported by several methods and promote genes strongly supported by a single approach (Equation S1). The *SSI* value was then corrected for the gene length (retrieved from Ensembl) [[30](#_ENREF_30)] where this strongly departed from the mean. The theoretical maximal *SSI* for an average-sized gene reported by all studies analyzed is 1, while genes reported by all studies within one methodological category would score 0.25 (Table S1). Thus, *SSI* weighs, combines and evaluates signals of selection on a per-gene basis, starting from the results of published genome-wide selection scans of autosomal loci. The list of top protein coding genes and their *SSI* values can be found in Additional file 5.

**Equation S1.** To compute a Selection Support Index (*SSI*) for each gene *i* with length *len_i_*, suppose *i* ∈ {1, 2, …, *n*}, and let *Diff_i_*, *LD_i_*, *SFS_i_* and *Comp_i_* be its selection supports within each methodological category across all compiled genome-wide selection scans. Gene length is measured in base pairs.

$$\mu=\frac{1}{n}\sum_{i=1}^{n} {len}_{i}$$

$${SSI}_{i}=\frac{{Diff}_{i}^{2}+{LD}_{i}^{2}+{SFS}_{i}^{2}+{Comp}_{i}^{2}}{4}\times\sqrt[10]{\frac{\mu}{{len}_{i}}}$$

**Table S1.** Selection support index values calculated for different scenarios.

|  | *Diff* | *LD* | *SFS* | *Comp* | *SSI* |
| --- | --- | --- | --- | --- | --- |
| *gene_1_* | 1 | 1 | 1 | 1 | 1 |
| *gene_2_* | 1 | 0 | 0 | 0 | 0.25 |
| *gene_3_* | 0.25 | 0.25 | 0.25 | 0.25 | 0.0625 |
| *…* |  |  |  |  |  |
| *gene_i_* | *Diff_i_* ∈ [0,1] | *LD_i_* ∈ [0,1] | *SFS_i_* ∈ [0,1] | *Comp_i_* ∈ [0,1] | *SSI_i_* ∈ [0,1] |
| *…* |  |  |  |  |  |
| *gene_n_* |  |  |  |  |  |

*gene_1_* – gene maximally supported by all methods; *gene_2_* – gene supported strongly by population differentiation methods only; *gene_3_* – gene poorly supported by all methods.

***Candidate selected genes from published surveys***

We assessed the confidence in selection on genes by an *in silico* quantification of the strength of the signal and its reproducibility in the meta-analysis. The most extreme selection events should leave the strongest signals, detectable by different methods, and thus be characterized by high reproducibility across independent studies. Although the ultimate goal of our analysis is to narrow down the signal of selection to a single causative variant, many selection scans identify large genomic regions and do not pinpoint a single causative SNP [[2](#_ENREF_2)]. Moreover, such scans often report outlier genes exhibiting the most extreme hallmarks of selection, instead of the precise genomic location of the signal itself. To nevertheless benefit from the rich data resource accumulated in the literature, we unified the selection-scan results by bringing them to the gene level and applying a per-gene ‘selection support index’ (*SSI* –Equation S1).

If classic hard sweeps were frequent in human evolution, we would find many candidate genes showing multiple signatures of selection and thus scoring highly in the meta-analysis. Instead, in agreement with previous meta-analyses [[1](#_ENREF_1), [14](#_ENREF_14), [31-36](#_ENREF_31)], we found many candidate genes that were reported by only one or a few studies, to which our index assigned low confidence in their selection (Additional file 1: Fig. S21.A). In contrast, some widely-accepted cases of adaptations with compelling functional evidence were found among our top-scoring candidates, such as *EDAR* [[37](#_ENREF_37), [38](#_ENREF_38)], *SLC24A5* [[39](#_ENREF_39)], *LCT*/*MCM6* [[40](#_ENREF_40), [41](#_ENREF_41)], *HERC2* and *OCA2* [[42-44](#_ENREF_42)]. On the other hand, the zinc uptake transporter ZIP4, known for its striking selection signature, did not show up among the top candidate genes in the meta-analysis of the published literature (Additional file 1: Fig. S21.A). ZIP4, encoded by *SLC39A4* is characterized by an extreme difference in the frequency of leucine-to-valine substitution (Leu372Val) between West Africans and Eurasians [[45](#_ENREF_45)]. The functionality of this variant was verified through *in vitro* functional experiments demonstrating differences between the human derived and ancestral alleles in surface protein expression, intracellular levels of zinc and zinc uptake [[45](#_ENREF_45)]. However, genomic scans for selection based on extended long haplotypes or deviations in the allele frequency spectrum had failed to identify ZIP4 as a candidate gene for positive selection. Such an extreme pattern of population differentiation and the absence of additional accompanying classic sweep signatures can be explained by the effect of a local recombination hotspot [[45](#_ENREF_45)]. In this scenario, *SLC39A4* should have obtained moderate support in our meta-analysis but was missed in many studies employing population differentiation methods, as the selected SNP (or any SNP tagging it) was not included in the commonly-used Affymetrix and Illumina SNP arrays and consequently it was absent from the HGDP and Perlegen datasets [[46](#_ENREF_46), [47](#_ENREF_47)]. As a result, *SLC39A4* was very weakly supported in our meta-analysis (Additional file 1: Fig. S21.A), although the selected variant was successfully picked up by *FineMAV* as one of the gold standards (Figure 4.A).

Even when a candidate gene has strong support from our index, rapid hard sweeps can result in a cluster of adjacent genes scoring highly (Additional file 1: Fig. S21.B) representing a single selection event spanning up to 1 Mb (e.g. the selection signal underlying lactose tolerance in Europeans which is detectable across a 1.3 Mb window as lactase (*LCT*)-surrounding genes are often reported as extreme outliers in selection studies (Additional file 1: Fig. S21.C)). The proportion of clustered candidate genes whose selection footprint could be explained by selection acting on a nearby gene depends on the *SSI* cutoff and varies from 50% up to 70% for top candidate selected genes (meeting the threshold of ≥ 0.17 (top ~1.5%) and ≥ 0.09 (top ~6%) respectively). However, we cannot exclude the possibility that in some cases selection truly acted on more than one gene within a cluster. Although the combined scans highlight potentially interesting signals, further functional validation is crucial for a signal to be considered real. To do so, the signature of selection needs to be narrowed down to one or a few candidate SNPs.

**Top *FineMAV* hits classification and enrichment analysis**

Our list of the top 300 candidates identified by *FineMAV* was annotated using Ensembl [[30](#_ENREF_30)] and was significantly enriched for variants of functional classes like missense mutations (p-value < 2.2 x 10^-16^, Fisher's Exact Test) or regulatory region variants (p-value = 5.30 x 10^-9^, Fisher's Exact Test) as compared to random expectation (assessed from a list of random alleles matched for the global allele frequency) (Additional file 1: Fig. S10). This is expected because of the inclusion of the *CADD* value [[48](#_ENREF_48)] in the *FineMAV* score.

We also used other measures of functionality to test our results, and observed that our outliers have higher *fitCons* scores (probability that a point mutation will influence fitness) [[49](#_ENREF_49)] (p-value < 2.2 x 10^-16^, Wilcoxon rank sum test) than expected by chance. Furthermore, variants falling in broadly non-functional classes (noncoding variation) are also are biased toward higher *GWAVA* scores (predicted functional impact of non-coding genetic variants) [[50](#_ENREF_50)] as compared with random expectation (p-value < 2.2 x 10^-16^, Wilcoxon rank sum test). These analyses were performed after excluding *FineMAV* hits on the sex chromosomes as *GWAVA* and *fitCons* scores are available for autosomes only [[49](#_ENREF_49), [50](#_ENREF_50)]. Thus although we used one particular measure of functionality in our discovery process, we also see very strong enrichment in other available functional prediction scores, which illustrates the consistency of our results.

We used the results of the meta-analysis of previous selection scans to compare *FineMAV* top hits with previous work. Our outliers fell in or near genes (~200 distinct genes) significantly enriched for high *SSI* from the meta-analysis, as compared to random expectation (p-value = 6.59 x 10^-10^, Wilcoxon rank sum test; after excluding gold standards: p-value = 9.20 x 10^-9^). This illustrates significant concordance with previous studies, as we find our strongest signals enriched in regions that have been independently identified as being under selection, although this comparison was limited to variants falling in or near genic regions on autosomes, as previous selection scans often do not report intergenic signals and excluded the sex chromosomes. We also compared the distribution of *FineMAV* scores of top SNPs falling in *SSI* outlier genes with the null expectation (top SNPs falling in matched random genes). To do so, we took the top ~1% of genes with the highest *SSI* scores (*SSI* ≥ 0.18), extended those genomic regions by 50 kb up- and downstream, extracted a top SNP falling in each window, and built a *FineMAV* distribution. We found this to be significantly different from the null expectation (p-value < 1.89 x 10^-5^) (Additional file 1: Fig. S22).

Additionally, we looked at the enrichment of GWAS hits among *FineMAV* outliers. Such comparison was carried out in LD framework instead of a simple overlap between *FineMAV* top SNPs and GWAS hits, as many GWAS studies rely on array-genotyping, rather than whole genome sequencing data and are confounded by LD between functional and linked variants. We therefore extracted the nearest up and downstream GWAS hit for each *FineMAV* outlier SNP using the GWAS catalogue (as our outliers could be tagged by nearby array-SNPs) and examined the linkage between each pair. We then looked at the enrichment of GWAS signal among our top outliers as compared to random matched SNPs. The enrichment was calculated for different linkage categories ranging from moderate to high LD (r^2^ ≥ 0.4)(Fisher's Exact Test). Sex chromosomes were excluded in this analysis as most of GWAS studies exclude them. We do see enrichment in GWAS hits among *FineMAV* outliers in Europeans and Eurasians (especially in the high LD category r^2^ ≥ 0.9) (Table S2). As the majority of GWAS studies were conducted in European-ancestry populations, the lack of enrichment in Africans and East Asians is not surprising.

Finally, we examined the overlap of *FineMAV* top hits and eQTL variants described in GTEx portal (as it offers eQTL annotation across various tissues) [[51](#_ENREF_51)]. Similarly to the above, we see an enrichment in eQTLs among *FineMAV* top outliers in Europeans and Eurasians as compared to random expectation (p-values of 0.04 and 0.03, respectively), although of lower significance than in case of GWAS signal (it is worth noting that eQTL analysis are limited to a limited selection of human tissues). In a similar fashion, non-European ancestries are underrepresented in eQTL databases. Over a half of the top 100 *FineMAV* outliers in Europeans and Eurasians were annotated as significant eQTLs (as indicated in Additional file 3).

**Table S2.** Enrichment in GWAS hits among *FineMAV* top outliers.

| Scenario | r^2^≥0.4 | r^2^≥0.5 | r^2^≥0.6 | r^2^≥0.7 | r^2^≥0.8 | r^2^≥0.9 |
| --- | --- | --- | --- | --- | --- | --- |
| AFR | 0.438 | 0.445 | 0.187 | 0.1 | 0.153 | 0.08 |
| EAS | 0.057 | 0.056 | 0.097 | 0.097 | 0.039 | 0.057 |
| EAS+EUR | 0.006 | 0.005 | 0.011 | 0.004 | 0.022 | 0.014 |
| EUR | 0.065 | 0.037 | 0.032 | 0.008 | 0.011 | 0.006 |

Significance of the enrichment (p-value) is given for each population (AFR – Africans; EAS – East Asians; EAS+EUR – Eurasians; EUR - Europeans) across different LD categories.

**Characterisation of chosen novel candidate variants**

***Nonsense variants***

We observed some high-scoring nonsense variants among our top candidates, suggesting pseudogenization of *PKD1L2* (an endogenous fatty acid synthase in skeletal muscle) [[52](#_ENREF_52)] in Europeans, *ZNF208* (zinc finger and SRY-interacting protein) [[53](#_ENREF_53)] in Africans, as well as *ZAN*, *OBSCN* (sacromeric signaling protein involved in myofibrillogenesis) [[54](#_ENREF_54)] and *MAGEE2* (melanoma-associated antigen expressed in the brain) [[51](#_ENREF_51)] in East Asians. Mice homozygous for knockout alleles of *OBSCN* and *ZAN* are viable and fertile [[55](#_ENREF_55), [56](#_ENREF_56)]; *ZAN* is particularly interesting as it encodes a zonadhesin protein located in the acrosome that mediates the species specificity of sperm binding to the extracellular coat of the egg (zona pellucida) [[57](#_ENREF_57)]. Sperm from zonadhesin-null mice exhibit dramatically higher levels of inter-species gamete adhesion without alteration in fertility [[56](#_ENREF_56)]. Zonadhesin is reported to be a rapidly-evolving protein with a high level of divergence between closely-related species, but is similar in species capable of interbreeding [[58](#_ENREF_58), [59](#_ENREF_59)]. The adaptive advantage of species specificity conferred by zonadhesin might be the limitation of cross-species fertilization and avoidance of sterile hybrids [[59](#_ENREF_59)]. However, polymorphism data in humans reveal a signature of positive selection in haplotypes carrying a frameshift mutation [[58](#_ENREF_58)]. We find a signal of selection at a nonsense mutation (rs2293766) present at 51% frequency in East Asians, but virtually absent elsewhere. An even higher frequency difference is observed for the stop allele at rs1343879 in *MAGEE2* on the X chromosome. Selection at this locus was previously reported by Yngvadottir et al., who observed lower diversity in haplotypes carrying the stop allele than in the others and concluded that like *ZAN,* MAGEE2 loss conferred a selective advantage in East Asia [[60](#_ENREF_60)].

***Missense variants***

*FineMAV* also highlighted rs6048066, a missense variant in *TGM3* in Africans. The *TGM3* gene product (TGase 3) is involved in the keratinization of the epidermis and hair follicle by crosslinking structural proteins, thereby contributing to hair structure, epidermal barrier functions and wound healing [[61](#_ENREF_61), [62](#_ENREF_62)]. *Tgm3* knockout mice do not exhibit severe malformation apart from striking abnormalities of hair follicle function and hair development, manifested by rough-looking, curly or brittle hair [[62-64](#_ENREF_62)]. The missense variant we report here falls in the catalytic core of the protein, as does the mouse nonsynonymous *we^Bkr^* allele causing a wavy coat and curly whisker phenotype [[64](#_ENREF_64)]. The absence of TGase 3 seems to affect hair fiber morphogenesis, and could play a role in the maintenance of body heat in mammals [[65](#_ENREF_65)]. Similarly in humans, TGase 3 is likely to participate in human hair shaft keratinization and scaffolding [[61](#_ENREF_61)], and its deficiency was linked to Uncombable Hair Syndrome characterized by dry, frizzy and wiry hair, often with slower hair growth rate [[66](#_ENREF_66)]. SNPs in *TGM3* have been weakly associated with hair diameter in humans [[67](#_ENREF_67)], and proteomic profiling of human hair shafts identified TGase 3 as a major component of the hair fiber and revealed considerable variation among samples of different ethnic origins, with the lowest levels in African Americans and Kenyans [[68](#_ENREF_68)]. We propose that this missense variant (rs6048066) might cause enzyme deficiency and contribute to African hair texture, hypothesized to have experienced strong positive selection in equatorial climates due to body-temperature-regulation [[69](#_ENREF_69), [70](#_ENREF_70)].

Another novel signal detected in African populations falls in *SPTA1*, encoding erythrocytic spectrin, alpha 1, a principal component of the erythrocyte membrane skeleton, which is essential for the arrangement of transmembrane proteins, determining red cell membrane stability, cell shape and deformability [[71-73](#_ENREF_71)]. Variants in *SPTA1* have been associated with quantitative hematologic traits [[74-76](#_ENREF_74)], and those causing its deficiency result in hemolytic anemias characterized by elliptically shaped erythrocytes (also seen in *Spta1^-/-^* null-mice) [[77](#_ENREF_77), [78](#_ENREF_78)]. The high prevalence of such anemia in Africa (10 times higher in West Africa than in Europe or USA) [[79](#_ENREF_79)] raised the question of a selective advantage, possibly contributing to protection against malaria [[80](#_ENREF_80), [81](#_ENREF_81)]. It has been shown that a decreased spectrin level inhibited malaria parasite growth *in vitro* [[82](#_ENREF_82)] and in a mouse model [[83](#_ENREF_83)]. This evidence suggests that a functionally and structurally normal host membrane is necessary for parasite growth and development [[80](#_ENREF_80), [82](#_ENREF_82)]. *FineMAV* pinpointed rs7547313 (Ile>Val) as a likely selected variant present at 0.37 frequency in Africans but absent elsewhere. However, the functional effect of this missense variant on the protein level and malaria parasite growth remains unknown.

***Regulatory variants***

Regulatory variants are particularly interesting as they form the most abundant functional category among *FineMAV* outliers and are responsible for the bulk of human phenotypic variation [[40](#_ENREF_40), [84](#_ENREF_84), [85](#_ENREF_85)]. However, the functional effect of regulatory variants remains difficult to predict and interpret. We find a signal of selection on rs2303893 - a splice region intronic regulatory variant that falls in a region flanking the *HADHB* promoter [[30](#_ENREF_30)] and is associated with increased *HADHB* expression in adipose, arterial and brain tissue in Geuvadis and GTEx data [[51](#_ENREF_51), [86](#_ENREF_86)]. *HADHB* encodes the beta subunit of the mitochondrial trifunctional protein involved in the beta-oxidation of fatty acids, and its deficiency causes severe phenotypes [[87-89](#_ENREF_87)], but the reason for selection in East Asians remains unknown.

Another candidate selected in East Asians is rs2224442, falling in a promoter-flanking region in the intron of *VRK1*. The region surrounding rs2224442, although non-coding, is characterized by high conservation across taxa and the presence of DNaseI hypersensitivity. VRK1 is a protein kinase implicated in mitotic and meiotic cell cycles, cell proliferation and differentiation [[90-93](#_ENREF_90)] that plays an important role in the organogenesis of sex organs and gametogenesis in multiple species [[94-97](#_ENREF_94)]. VRK1-deficient organisms show abnormality of the reproductive organs, followed by defects in germ cell development [[94-97](#_ENREF_94)]. Both sexes of VRK1-null mice have been reported to be infertile displaying defects in sex organs (e.g. small testis in male) and impaired oogenesis and spermatogenesis due to meiotic arrest manifested as azoospermia and lack of mature sperm in males [[98-101](#_ENREF_98)]. It seems possible that this regulatory variant affects the expression level of *VRK1* and modulates the maturation of gametes.

**References**

1. Akey JM: **Constructing genomic maps of positive selection in humans: where do we go from here?** *Genome Res* 2009, **19:**711-722.

2. Vitti JJ, Grossman SR, Sabeti PC: **Detecting natural selection in genomic data.** *Annu Rev Genet* 2013, **47:**97-120.

3. Akey JM, Zhang G, Zhang K, Jin L, Shriver MD: **Interrogating a high-density SNP map for signatures of natural selection.** *Genome Res* 2002, **12:**1805-1814.

4. Liu X, Ong RT, Pillai EN, Elzein AM, Small KS, Clark TG, Kwiatkowski DP, Teo YY: **Detecting and characterizing genomic signatures of positive selection in global populations.** *Am J Hum Genet* 2013, **92:**866-881.

5. Barreiro LB, Laval G, Quach H, Patin E, Quintana-Murci L: **Natural selection has driven population differentiation in modern humans.** *Nat Genet* 2008, **40:**340-345.

6. Carlson CS, Thomas DJ, Eberle MA, Swanson JE, Livingston RJ, Rieder MJ, Nickerson DA: **Genomic regions exhibiting positive selection identified from dense genotype data.** *Genome Res* 2005, **15:**1553-1565.

7. Chen H, Patterson N, Reich D: **Population differentiation as a test for selective sweeps.** *Genome Res* 2010, **20:**393-402.

8. International HapMap C, Frazer KA, Ballinger DG, Cox DR, Hinds DA, Stuve LL, Gibbs RA, Belmont JW, Boudreau A, Hardenbol P, et al: **A second generation human haplotype map of over 3.1 million SNPs.** *Nature* 2007, **449:**851-861.

9. Fagny M, Patin E, Enard D, Barreiro LB, Quintana-Murci L, Laval G: **Exploring the occurrence of classic selective sweeps in humans using whole-genome sequencing data sets.** *Mol Biol Evol* 2014, **31:**1850-1868.

10. Grossman SR, Shlyakhter I, Karlsson EK, Byrne EH, Morales S, Frieden G, Hostetter E, Angelino E, Garber M, Zuk O, et al: **A composite of multiple signals distinguishes causal variants in regions of positive selection.** *Science* 2010, **327:**883-886.

11. Hofer T, Foll M, Excoffier L: **Evolutionary forces shaping genomic islands of population differentiation in humans.** *BMC Genomics* 2012, **13:**107.

12. Tennessen JA, Akey JM: **Parallel adaptive divergence among geographically diverse human populations.** *PLoS Genet* 2011, **7:**e1002127.

13. Johansson A, Gyllensten U: **Identification of local selective sweeps in human populations since the exodus from Africa.** *Hereditas* 2008, **145:**126-137.

14. Kelley JL, Madeoy J, Calhoun JC, Swanson W, Akey JM: **Genomic signatures of positive selection in humans and the limits of outlier approaches.** *Genome Res* 2006, **16:**980-989.

15. Kimura R, Fujimoto A, Tokunaga K, Ohashi J: **A practical genome scan for population-specific strong selective sweeps that have reached fixation.** *PLoS One* 2007, **2:**e286.

16. Lopez Herraez D, Bauchet M, Tang K, Theunert C, Pugach I, Li J, Nandineni MR, Gross A, Scholz M, Stoneking M: **Genetic variation and recent positive selection in worldwide human populations: evidence from nearly 1 million SNPs.** *PLoS One* 2009, **4:**e7888.

17. Pickrell JK, Coop G, Novembre J, Kudaravalli S, Li JZ, Absher D, Srinivasan BS, Barsh GS, Myers RM, Feldman MW, Pritchard JK: **Signals of recent positive selection in a worldwide sample of human populations.** *Genome Res* 2009, **19:**826-837.

18. Rafajlovic M, Klassmann A, Eriksson A, Wiehe T, Mehlig B: **Demography-adjusted tests of neutrality based on genome-wide SNP data.** *Theor Popul Biol* 2014, **95:**1-12.

19. Sabeti PC, Varilly P, Fry B, Lohmueller J, Hostetter E, Cotsapas C, Xie X, Byrne EH, McCarroll SA, Gaudet R, et al: **Genome-wide detection and characterization of positive selection in human populations.** *Nature* 2007, **449:**913-918.

20. Grossman SR, Andersen KG, Shlyakhter I, Tabrizi S, Winnicki S, Yen A, Park DJ, Griesemer D, Karlsson EK, Wong SH, et al: **Identifying recent adaptations in large-scale genomic data.** *Cell* 2013, **152:**703-713.

21. Voight BF, Kudaravalli S, Wen X, Pritchard JK: **A map of recent positive selection in the human genome.** *PLoS Biol* 2006, **4:**e72.

22. Cai Z, Camp NJ, Cannon-Albright L, Thomas A: **Identification of regions of positive selection using Shared Genomic Segment analysis.** *Eur J Hum Genet* 2011, **19:**667-671.

23. Zhong M, Lange K, Papp JC, Fan R: **A powerful score test to detect positive selection in genome-wide scans.** *Eur J Hum Genet* 2010, **18:**1148-1159.

24. Tang K, Thornton KR, Stoneking M: **A new approach for using genome scans to detect recent positive selection in the human genome.** *PLoS Biol* 2007, **5:**e171.

25. Wang ET, Kodama G, Baldi P, Moyzis RK: **Global landscape of recent inferred Darwinian selection for Homo sapiens.** *Proc Natl Acad Sci U S A* 2006, **103:**135-140.

26. Williamson SH, Hubisz MJ, Clark AG, Payseur BA, Bustamante CD, Nielsen R: **Localizing recent adaptive evolution in the human genome.** *PLoS Genet* 2007, **3:**e90.

27. Zhang C, Bailey DK, Awad T, Liu G, Xing G, Cao M, Valmeekam V, Retief J, Matsuzaki H, Taub M, et al: **A whole genome long-range haplotype (WGLRH) test for detecting imprints of positive selection in human populations.** *Bioinformatics* 2006, **22:**2122-2128.

28. Colonna V, Ayub Q, Chen Y, Pagani L, Luisi P, Pybus M, Garrison E, Xue Y, Tyler-Smith C, Genomes Project C, et al: **Human genomic regions with exceptionally high levels of population differentiation identified from 911 whole-genome sequences.** *Genome Biol* 2014, **15:**R88.

29. Genomes Project C, Abecasis GR, Auton A, Brooks LD, DePristo MA, Durbin RM, Handsaker RE, Kang HM, Marth GT, McVean GA: **An integrated map of genetic variation from 1,092 human genomes.** *Nature* 2012, **491:**56-65.

30. Cunningham F, Amode MR, Barrell D, Beal K, Billis K, Brent S, Carvalho-Silva D, Clapham P, Coates G, Fitzgerald S, et al: **Ensembl 2015.** *Nucleic Acids Res* 2015, **43:**D662-669.

31. Pritchard JK, Pickrell JK, Coop G: **The genetics of human adaptation: hard sweeps, soft sweeps, and polygenic adaptation.** *Curr Biol* 2010, **20:**R208-215.

32. Hernandez RD, Kelley JL, Elyashiv E, Melton SC, Auton A, McVean G, Genomes P, Sella G, Przeworski M: **Classic selective sweeps were rare in recent human evolution.** *Science* 2011, **331:**920-924.

33. Nielsen R, Hellmann I, Hubisz M, Bustamante C, Clark AG: **Recent and ongoing selection in the human genome.** *Nat Rev Genet* 2007, **8:**857-868.

34. Pavlidis P, Jensen JD, Stephan W, Stamatakis A: **A critical assessment of storytelling: gene ontology categories and the importance of validating genomic scans.** *Mol Biol Evol* 2012, **29:**3237-3248.

35. Biswas S, Akey JM: **Genomic insights into positive selection.** *Trends Genet* 2006, **22:**437-446.

36. Oleksyk TK, Smith MW, O'Brien SJ: **Genome-wide scans for footprints of natural selection.** *Philos Trans R Soc Lond B Biol Sci* 2010, **365:**185-205.

37. Kamberov YG, Wang S, Tan J, Gerbault P, Wark A, Tan L, Yang Y, Li S, Tang K, Chen H, et al: **Modeling recent human evolution in mice by expression of a selected EDAR variant.** *Cell* 2013, **152:**691-702.

38. Mou C, Thomason HA, Willan PM, Clowes C, Harris WE, Drew CF, Dixon J, Dixon MJ, Headon DJ: **Enhanced ectodysplasin-A receptor (EDAR) signaling alters multiple fiber characteristics to produce the East Asian hair form.** *Hum Mutat* 2008, **29:**1405-1411.

39. Lamason RL, Mohideen MA, Mest JR, Wong AC, Norton HL, Aros MC, Jurynec MJ, Mao X, Humphreville VR, Humbert JE, et al: **SLC24A5, a putative cation exchanger, affects pigmentation in zebrafish and humans.** *Science* 2005, **310:**1782-1786.

40. Enattah NS, Sahi T, Savilahti E, Terwilliger JD, Peltonen L, Jarvela I: **Identification of a variant associated with adult-type hypolactasia.** *Nat Genet* 2002, **30:**233-237.

41. Olds LC, Sibley E: **Lactase persistence DNA variant enhances lactase promoter activity in vitro: functional role as a cis regulatory element.** *Hum Mol Genet* 2003, **12:**2333-2340.

42. Sturm RA, Duffy DL, Zhao ZZ, Leite FP, Stark MS, Hayward NK, Martin NG, Montgomery GW: **A single SNP in an evolutionary conserved region within intron 86 of the HERC2 gene determines human blue-brown eye color.** *Am J Hum Genet* 2008, **82:**424-431.

43. Eiberg H, Troelsen J, Nielsen M, Mikkelsen A, Mengel-From J, Kjaer KW, Hansen L: **Blue eye color in humans may be caused by a perfectly associated founder mutation in a regulatory element located within the HERC2 gene inhibiting OCA2 expression.** *Hum Genet* 2008, **123:**177-187.

44. Visser M, Kayser M, Palstra RJ: **HERC2 rs12913832 modulates human pigmentation by attenuating chromatin-loop formation between a long-range enhancer and the OCA2 promoter.** *Genome Res* 2012, **22:**446-455.

45. Engelken J, Carnero-Montoro E, Pybus M, Andrews GK, Lalueza-Fox C, Comas D, Sekler I, de la Rasilla M, Rosas A, Stoneking M, et al: **Extreme population differences in the human zinc transporter ZIP4 (SLC39A4) are explained by positive selection in Sub-Saharan Africa.** *PLoS Genet* 2014, **10:**e1004128.

46. Li JZ, Absher DM, Tang H, Southwick AM, Casto AM, Ramachandran S, Cann HM, Barsh GS, Feldman M, Cavalli-Sforza LL, Myers RM: **Worldwide human relationships inferred from genome-wide patterns of variation.** *Science* 2008, **319:**1100-1104.

47. Peacock E, Whiteley P: **Perlegen sciences, inc.** *Pharmacogenomics* 2005, **6:**439-442.

48. Kircher M, Witten DM, Jain P, O'Roak BJ, Cooper GM, Shendure J: **A general framework for estimating the relative pathogenicity of human genetic variants.** *Nat Genet* 2014, **46:**310-315.

49. Gulko B, Hubisz MJ, Gronau I, Siepel A: **A method for calculating probabilities of fitness consequences for point mutations across the human genome.** *Nat Genet* 2015, **47:**276-283.

50. Ritchie GR, Dunham I, Zeggini E, Flicek P: **Functional annotation of noncoding sequence variants.** *Nat Methods* 2014, **11:**294-296.

51. Consortium GT: **Human genomics. The Genotype-Tissue Expression (GTEx) pilot analysis: multitissue gene regulation in humans.** *Science* 2015, **348:**648-660.

52. Mackenzie FE, Romero R, Williams D, Gillingwater T, Hilton H, Dick J, Riddoch-Contreras J, Wong F, Ireson L, Powles-Glover N, et al: **Upregulation of PKD1L2 provokes a complex neuromuscular disease in the mouse.** *Hum Mol Genet* 2009, **18:**3553-3566.

53. Oh HJ, Li Y, Lau YF: **Sry associates with the heterochromatin protein 1 complex by interacting with a KRAB domain protein.** *Biol Reprod* 2005, **72:**407-415.

54. Young P, Ehler E, Gautel M: **Obscurin, a giant sarcomeric Rho guanine nucleotide exchange factor protein involved in sarcomere assembly.** *J Cell Biol* 2001, **154:**123-136.

55. Randazzo D, Giacomello E, Lorenzini S, Rossi D, Pierantozzi E, Blaauw B, Reggiani C, Lange S, Peter AK, Chen J, Sorrentino V: **Obscurin is required for ankyrinB-dependent dystrophin localization and sarcolemma integrity.** *J Cell Biol* 2013, **200:**523-536.

56. Tardif S, Wilson MD, Wagner R, Hunt P, Gertsenstein M, Nagy A, Lobe C, Koop BF, Hardy DM: **Zonadhesin is essential for species specificity of sperm adhesion to the egg zona pellucida.** *J Biol Chem* 2010, **285:**24863-24870.

57. Wassarman PM, Jovine L, Litscher ES: **A profile of fertilization in mammals.** *Nat Cell Biol* 2001, **3:**E59-64.

58. Gasper J, Swanson WJ: **Molecular population genetics of the gene encoding the human fertilization protein zonadhesin reveals rapid adaptive evolution.** *Am J Hum Genet* 2006, **79:**820-830.

59. Tardif S, Brady HA, Breazeale KR, Bi M, Thompson LD, Bruemmer JE, Bailey LB, Hardy DM: **Zonadhesin D3-polypeptides vary among species but are similar in Equus species capable of interbreeding.** *Biol Reprod* 2010, **82:**413-421.

60. Yngvadottir B, Xue Y, Searle S, Hunt S, Delgado M, Morrison J, Whittaker P, Deloukas P, Tyler-Smith C: **A genome-wide survey of the prevalence and evolutionary forces acting on human nonsense SNPs.** *Am J Hum Genet* 2009, **84:**224-234.

61. Thibaut S, Cavusoglu N, de Becker E, Zerbib F, Bednarczyk A, Schaeffer C, van Dorsselaer A, Bernard BA: **Transglutaminase-3 enzyme: a putative actor in human hair shaft scaffolding?** *J Invest Dermatol* 2009, **129:**449-459.

62. John S, Thiebach L, Frie C, Mokkapati S, Bechtel M, Nischt R, Rosser-Davies S, Paulsson M, Smyth N: **Epidermal transglutaminase (TGase 3) is required for proper hair development, but not the formation of the epidermal barrier.** *PLoS One* 2012, **7:**e34252.

63. Bognar P, Nemeth I, Mayer B, Haluszka D, Wikonkal N, Ostorhazi E, John S, Paulsson M, Smyth N, Pasztoi M, et al: **Reduced inflammatory threshold indicates skin barrier defect in transglutaminase 3 knockout mice.** *J Invest Dermatol* 2014, **134:**105-111.

64. Brennan BM, Huynh MT, Rabah MA, Shaw HE, Bisaillon JJ, Radden LA, 2nd, Nguyen TV, King TR: **The mouse wellhaarig (we) mutations result from defects in epidermal-type transglutaminase 3 (Tgm3).** *Mol Genet Metab* 2015, **116:**187-191.

65. Steinert PM, Parry DA, Marekov LN: **Trichohyalin mechanically strengthens the hair follicle: multiple cross-bridging roles in the inner root shealth.** *J Biol Chem* 2003, **278:**41409-41419.

66. FB UB, Cau L, Tafazzoli A, Mechin MC, Wolf S, Romano MT, Valentin F, Wiegmann H, Huchenq A, Kandil R, et al: **Mutations in Three Genes Encoding Proteins Involved in Hair Shaft Formation Cause Uncombable Hair Syndrome.** *Am J Hum Genet* 2016, **99:**1292-1304.

67. Fujimoto A, Nishida N, Kimura R, Miyagawa T, Yuliwulandari R, Batubara L, Mustofa MS, Samakkarn U, Settheetham-Ishida W, Ishida T, et al: **FGFR2 is associated with hair thickness in Asian populations.** *J Hum Genet* 2009, **54:**461-465.

68. Laatsch CN, Durbin-Johnson BP, Rocke DM, Mukwana S, Newland AB, Flagler MJ, Davis MG, Eigenheer RA, Phinney BS, Rice RH: **Human hair shaft proteomic profiling: individual differences, site specificity and cuticle analysis.** *PeerJ* 2014, **2:**e506.

69. Jablonski NG, Chaplin G: **The evolution of skin pigmentation and hair texture in people of African ancestry.** *Dermatol Clin* 2014, **32:**113-121.

70. Robbins CR: *Chemical and physical behavior of human hair.* 4th edn. New York: Springer; 2002.

71. Iolascon A, King MJ, Robertson S, Avvisati RA, Vitiello F, Asci R, Scoppettuolo MN, Delaunay J: **A genomic deletion causes truncation of alpha-spectrin and ellipto-poikilocytosis.** *Blood Cells Mol Dis* 2011, **46:**195-200.

72. Salomao M, An X, Guo X, Gratzer WB, Mohandas N, Baines AJ: **Mammalian alpha I-spectrin is a neofunctionalized polypeptide adapted to small highly deformable erythrocytes.** *Proc Natl Acad Sci U S A* 2006, **103:**643-648.

73. Burke JP, Van Zyl D, Zail SS, Coetzer TL: **Reduced spectrin-ankyrin binding in a South African hereditary elliptocytosis kindred homozygous for spectrin St Claude.** *Blood* 1998, **92:**2591-2592.

74. Soranzo N, Sanna S, Wheeler E, Gieger C, Radke D, Dupuis J, Bouatia-Naji N, Langenberg C, Prokopenko I, Stolerman E, et al: **Common variants at 10 genomic loci influence hemoglobin A(1)(C) levels via glycemic and nonglycemic pathways.** *Diabetes* 2010, **59:**3229-3239.

75. Ding K, Shameer K, Jouni H, Masys DR, Jarvik GP, Kho AN, Ritchie MD, McCarty CA, Chute CG, Manolio TA, Kullo IJ: **Genetic Loci implicated in erythroid differentiation and cell cycle regulation are associated with red blood cell traits.** *Mayo Clin Proc* 2012, **87:**461-474.

76. Ganesh SK, Zakai NA, van Rooij FJ, Soranzo N, Smith AV, Nalls MA, Chen MH, Kottgen A, Glazer NL, Dehghan A, et al: **Multiple loci influence erythrocyte phenotypes in the CHARGE Consortium.** *Nat Genet* 2009, **41:**1191-1198.

77. Birkenmeier CS, McFarland-Starr EC, Barker JE: **Chromosomal location of three spectrin genes: relationship to the inherited hemolytic anemias of mouse and man.** *Proc Natl Acad Sci U S A* 1988, **85:**8121-8125.

78. Grossmann A, Maggio-Price L, Shiota FM, Liggitt DV: **Pathologic features associated with decreased longevity of mutant sphha/sphha mice with chronic hemolytic anemia: similarities to sequelae of sickle cell anemia in humans.** *Lab Anim Sci* 1993, **43:**217-221.

79. Lecomte MC, Dhermy D, Gautero H, Bournier O, Galand C, Boivin P: **[Hereditary elliptocytosis in West Africa: frequency and repartition of spectrin variants].** *C R Acad Sci III* 1988, **306:**43-46.

80. Delaunay J, Dhermy D: **Mutations involving the spectrin heterodimer contact site: clinical expression and alterations in specific function.** *Semin Hematol* 1993, **30:**21-33.

81. Delaunay J: **The molecular basis of hereditary red cell membrane disorders.** *Blood Rev* 2007, **21:**1-20.

82. Schulman S, Roth EF, Jr., Cheng B, Rybicki AC, Sussman, II, Wong M, Wang W, Ranney HM, Nagel RL, Schwartz RS: **Growth of Plasmodium falciparum in human erythrocytes containing abnormal membrane proteins.** *Proc Natl Acad Sci U S A* 1990, **87:**7339-7343.

83. Shear HL, Roth EF, Jr., Ng C, Nagel RL: **Resistance to malaria in ankyrin and spectrin deficient mice.** *Br J Haematol* 1991, **78:**555-560.

84. Carroll SB: **Evo-devo and an expanding evolutionary synthesis: a genetic theory of morphological evolution.** *Cell* 2008, **134:**25-36.

85. Scheinfeldt LB, Tishkoff SA: **Recent human adaptation: genomic approaches, interpretation and insights.** *Nat Rev Genet* 2013, **14:**692-702.

86. Lappalainen T, Sammeth M, Friedlander MR, t Hoen PA, Monlong J, Rivas MA, Gonzalez-Porta M, Kurbatova N, Griebel T, Ferreira PG, et al: **Transcriptome and genome sequencing uncovers functional variation in humans.** *Nature* 2013, **501:**506-511.

87. Spiekerkoetter U, Sun B, Khuchua Z, Bennett MJ, Strauss AW: **Molecular and phenotypic heterogeneity in mitochondrial trifunctional protein deficiency due to beta-subunit mutations.** *Hum Mutat* 2003, **21:**598-607.

88. Purevsuren J, Fukao T, Hasegawa Y, Kobayashi H, Li H, Mushimoto Y, Fukuda S, Yamaguchi S: **Clinical and molecular aspects of Japanese patients with mitochondrial trifunctional protein deficiency.** *Mol Genet Metab* 2009, **98:**372-377.

89. Naiki M, Ochi N, Kato YS, Purevsuren J, Yamada K, Kimura R, Fukushi D, Hara S, Yamada Y, Kumagai T, et al: **Mutations in HADHB, which encodes the beta-subunit of mitochondrial trifunctional protein, cause infantile onset hypoparathyroidism and peripheral polyneuropathy.** *Am J Med Genet A* 2014, **164A:**1180-1187.

90. Valbuena A, Lopez-Sanchez I, Lazo PA: **Human VRK1 is an early response gene and its loss causes a block in cell cycle progression.** *PLoS One* 2008, **3:**e1642.

91. Valbuena A, Sanz-Garcia M, Lopez-Sanchez I, Vega FM, Lazo PA: **Roles of VRK1 as a new player in the control of biological processes required for cell division.** *Cell Signal* 2011, **23:**1267-1272.

92. Moura DS, Fernandez IF, Marin-Royo G, Lopez-Sanchez I, Martin-Doncel E, Vega FM, Lazo PA: **Oncogenic Sox2 regulates and cooperates with VRK1 in cell cycle progression and differentiation.** *Sci Rep* 2016, **6:**28532.

93. Liu J, Wang Y, He S, Xu X, Huang Y, Tang J, Wu Y, Miao X, He Y, Wang Q, et al: **Expression of vaccinia-related kinase 1 (VRK1) accelerates cell proliferation but overcomes cell adhesion mediated drug resistance (CAM-DR) in multiple myeloma.** *Hematology* 2016**:**1-10.

94. Lancaster OM, Breuer M, Cullen CF, Ito T, Ohkura H: **The meiotic recombination checkpoint suppresses NHK-1 kinase to prevent reorganisation of the oocyte nucleus in Drosophila.** *PLoS Genet* 2010, **6:**e1001179.

95. Waters K, Yang AZ, Reinke V: **Genome-wide analysis of germ cell proliferation in C.elegans identifies VRK-1 as a key regulator of CEP-1/p53.** *Dev Biol* 2010, **344:**1011-1025.

96. Dobrzynska A, Askjaer P: **Vaccinia-related kinase 1 is required for early uterine development in Caenorhabditis elegans.** *Dev Biol* 2016, **411:**246-256.

97. Ivanovska I, Khandan T, Ito T, Orr-Weaver TL: **A histone code in meiosis: the histone kinase, NHK-1, is required for proper chromosomal architecture in Drosophila oocytes.** *Genes Dev* 2005, **19:**2571-2582.

98. Kim J, Choi YH, Chang S, Kim KT, Je JH: **Defective folliculogenesis in female mice lacking Vaccinia-related kinase 1.** *Sci Rep* 2012, **2:**468.

99. Schober CS, Aydiner F, Booth CJ, Seli E, Reinke V: **The kinase VRK1 is required for normal meiotic progression in mammalian oogenesis.** *Mech Dev* 2011, **128:**178-190.

100. Choi YH, Park CH, Kim W, Ling H, Kang A, Chang MW, Im SK, Jeong HW, Kong YY, Kim KT: **Vaccinia-related kinase 1 is required for the maintenance of undifferentiated spermatogonia in mouse male germ cells.** *PLoS One* 2010, **5:**e15254.

101. Wiebe MS, Nichols RJ, Molitor TP, Lindgren JK, Traktman P: **Mice deficient in the serine/threonine protein kinase VRK1 are infertile due to a progressive loss of spermatogonia.** *Biol Reprod* 2010, **82:**182-193.
